# Autophagy as a Target for Drug Development Of Skin Infection Caused by Mycobacteria

**DOI:** 10.3389/fimmu.2021.674241

**Published:** 2021-05-25

**Authors:** Tamiris Lameira Bittencourt, Rhana Berto da Silva Prata, Bruno Jorge de Andrade Silva, Mayara Garcia de Mattos Barbosa, Margareth Pretti Dalcolmo, Roberta Olmo Pinheiro

**Affiliations:** ^1^ Leprosy Laboratory, Oswaldo Cruz Institute, Oswaldo Cruz Foundation (Fiocruz), Rio de Janeiro, Brazil; ^2^ Division of Dermatology, David Geffen School of Medicine, Los Angeles, CA, United States; ^3^ Department of Surgery, University of Michigan, Ann Arbor, MI, United States; ^4^ Helio Fraga Reference Center, Sergio Arouca National School of Public Health, Fiocruz, Rio de Janeiro, Brazil

**Keywords:** autophagy, skin, mycobacteria, drug development, skin cells

## Abstract

Pathogenic mycobacteria species may subvert the innate immune mechanisms and can modulate the activation of cells that cause disease in the skin. Cutaneous mycobacterial infection may present different clinical presentations and it is associated with stigma, deformity, and disability. The understanding of the immunopathogenic mechanisms related to mycobacterial infection in human skin is of pivotal importance to identify targets for new therapeutic strategies. The occurrence of reactional episodes and relapse in leprosy patients, the emergence of resistant mycobacteria strains, and the absence of effective drugs to treat mycobacterial cutaneous infection increased the interest in the development of therapies based on repurposed drugs against mycobacteria. The mechanism of action of many of these therapies evaluated is linked to the activation of autophagy. Autophagy is an evolutionary conserved lysosomal degradation pathway that has been associated with the control of the mycobacterial bacillary load. Here, we review the role of autophagy in the pathogenesis of cutaneous mycobacterial infection and discuss the perspectives of autophagy as a target for drug development and repurposing against cutaneous mycobacterial infection.

## Introduction

Pathogenic mycobacteria species subvert the innate immune system barriers and modulate the activation of phagocytes to cause disease not only in the respiratory tract but also in soft tissues and skin, sometimes resulting in disseminated infection (1). Cutaneous mycobacterial infections may cause different clinical manifestations, such as cutaneous manifestations of *Mycobacterium tuberculosis* (*M. tuberculosis*) infection, Buruli ulcer caused by *M. ulcerans* and other related slowly growing mycobacteria, leprosy caused by *M. leprae* and *M. lepromatosis*, and cutaneous infections caused by rapidly growing mycobacteria such as *M. abscessus* subsp. *abscessus*, *M. abscessus* subsp. *bolletti*, *M. abscessus* subsp. *massiliense*, *M. chelonae* and *M. fortuitum* (1–9). Among patients with advanced immunosuppression, *M. avium-intracellulare* complex, the *M. haemophilum*, and *M. kansasii* may cause cutaneous or disseminated disease. Mycobacterial infections of the skin and subcutaneous tissue are associated with important stigma, deformity, and disability. The treatment for cutaneous mycobacterial infections depends on the specific pathogen, whereas for rapidly growing mycobacteria, the official treatment guidelines recommend carrying out susceptibility tests for antibacterial drugs of different classes (10, 11). Management often includes use of multiple antibiotics for several months (12). Treatment options for cutaneous tuberculosis follow the same recommendations for the treatment of other forms of TB, being limited to conventional oral therapy and surgical intervention for severe forms, such as lupus vulgaris (13, 14). The therapeutic regimen is based on the combination of isoniazid, rifampicin, pyrazinamide, ethambutol and streptomycin according to the needs of each individual. In most cases, skin manifestations result from hematogenous dissemination or are a direct extension from the focus of the infection (14, 15). In addition, treatment of leprosy is performed with multidrug therapy (MDT) and comprises 6 or 12 doses, depending on the clinical form. There is not a consensus for the treatment of cutaneous infections caused by non-tuberculous mycobacteria. Recently, much effort has been made to develop more effective therapies by modulating host responses to mycobacteria (i.e., host-directed therapy).

After recognition by skin cells, mycobacteria may use a wide range of strategies to escape the microbicidal activity of skin host cells. Some of these immune escape mechanisms are the inhibition of the maturation of phagolysosomes, inhibition of the acidification of phagolysosomes, bacterial escape to reside in the cytosol, modulation of host cell metabolism, inhibition of oxidative stress, and inhibition of apoptosis and autophagy associated with increased type 1 interferon (IFN) expression and inflammasome activation (16–23).

Autophagy is an intracellular catabolic process that may contribute to the removal of invading pathogens *via* a lysosomal degradation pathway. The activation of autophagy by diverse drugs or agents may represent a promising treatment strategy against mycobacterial diseases. In this review, we discuss the current knowledge of, advances and perspectives on new therapeutic strategies targeting autophagy against mycobacterial infections in the skin.

## Overview of Autophagy Machinery on Skin Cells

Autophagy is a homeostatic mechanism highly conserved evolutionarily and dependent on the lysosome action (24). It is responsible for the cellular catabolism of dysfunctional organelles, components of the cytoplasm and, more recently, invading pathogens, thus determining the maintenance of homeostasis and adaptation of the cell to stress (25, 26). Autophagy has been described as having a primary role in physiological cellular processes such as development and growth, in the senescence process, and immune defense (25, 27–29). Based on the way the autophagy target is taken to the lysosome, its final destination of degradation, autophagy was didactically classified into three forms: macroautophagy, microautophagy, and chaperone-mediated autophagy. In this review, we will exclusively address the action and manipulation of the macroautophagy pathway.

Only a small amount of research has considered the impact of autophagy on the pathogenesis of skin diseases, including diseases caused by mycobacteria. Skin is the largest organ of the body and it is not only the first line of defense against numerous insults but it is also the site whereas some infectious, including mycobacterial diseases, may manifest.

Autophagy is considered an effector tool of the immune system since it is a relevant pathway of elimination and recognition of pathogens by the immune system (30). As well to cellular homeostasis, autophagy works to eliminate intracellular pathogens, including some pathogens associated with skin diseases, such as *Streptococcus pyogenes* from group A (31, 32), *Staphylococcus aureus* (33, 34), *M. leprae* (35, 36), *M. marinum* (37, 38), and *M. tuberculosis* (39–42). Through a process called xenophagy, which plays a principal role in innate immune defense, intracellular pathogens are directed to the autophagosome and then to the lysosomal degradation pathway (43, 44). Xenophagy is the process of eliminating intracellular pathogens through autophagic machinery, being a unique type of macroautophagy/selective autophagy that targets invasive pathogens, being an important defense mechanism against infectious diseases (45, 46).

Few studies have focused on deciphering autophagy machinery in skin cells, such as: keratinocytes, skin fibroblasts, melanocytes, Langerhans cells, dendritic cells, mast cells, neutrophils, NK and B cells. The current knowledge regarding skin cell autophagy during mycobacterial diseases is based mainly in studies with cell lineage and dermal macrophages.

**Figure 1:**
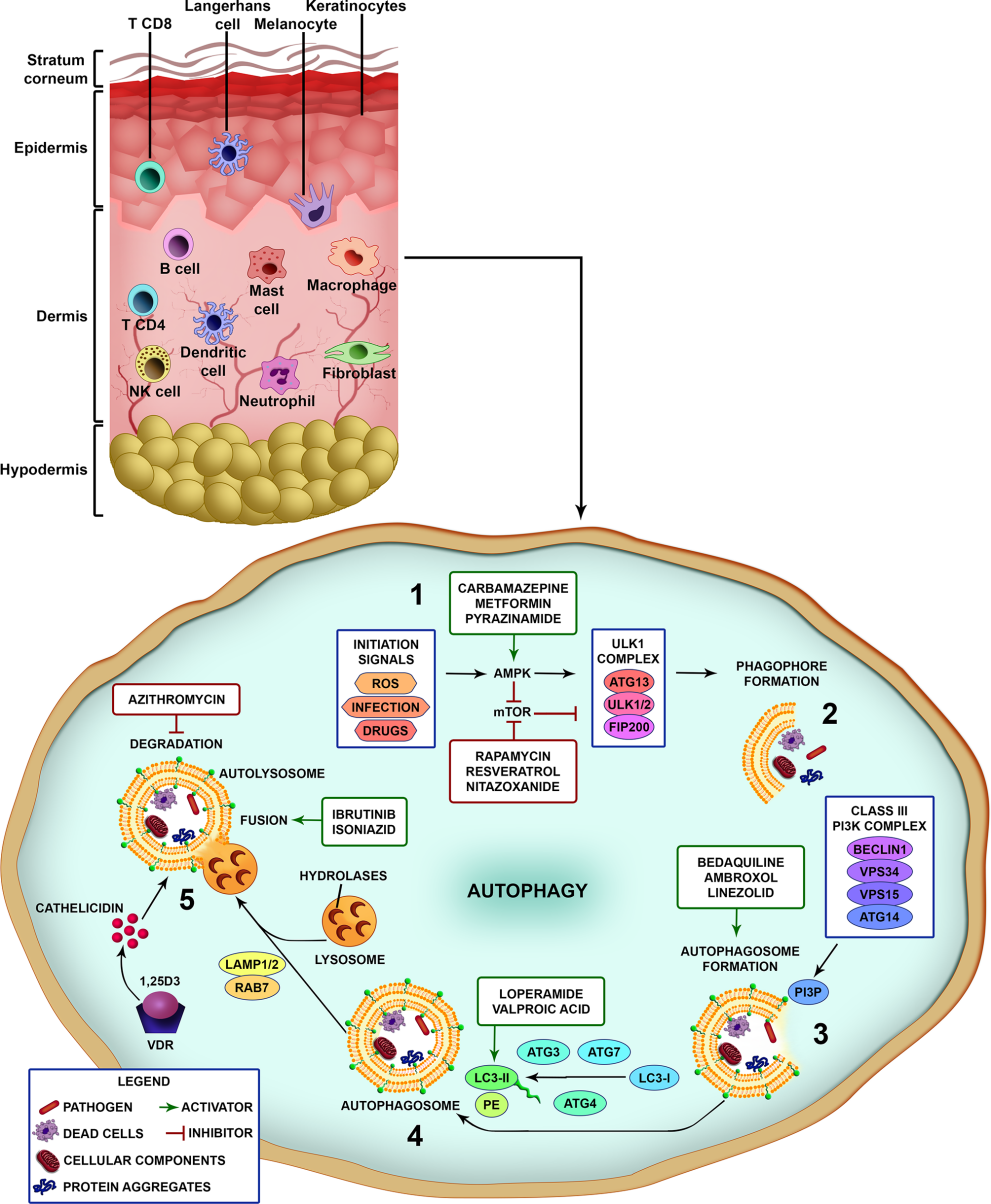
Different steps of the autophagic pathway targeted by autophagy-modulating drugs. A schematic view of the different cell types populating the skin. Vertebrate skin is comprised of two major compartments: the epidermis and the dermis. The superficial part of the epidermis, known as the stratum corneum, is composed of dead keratinocytes and acts as a barrier. The epidermis is composed mainly of keratinocytes with few melanocytes. The major immune cells in this compartment include Langerhans cells (LCs) and CD8 T-cells. The dermis is composed of fibroblasts, NK cells, T-cells (CD4 αβ, and γδ), B cells, dermal dendritic cells, macrophages, mast cells, and neutrophils (non-exhaustive list). The knowledge of skin cell autophagy is mainly based in studies with dermal macrophages. Briefly, (1) autophagy is inhibited by mTOR and activated by AMPK. mTOR is inhibited by the autophagy-initiation signals as metabolic stress, ROS, infection and drugs, and leads to the activation of AMPK. After AMPK activation, the ULK1 complex (ATG13, ULK1/2, FIP200) initiates the phagophore formation (2), involving the targets (pathogens, dead cells, cellular components and organelles, protein aggregates), which in turn activates the Class III PI3K complex (Beclin 1, VPS34, VPS15, ATG14) (3). This complex completes the autophagosome maturation and elongation by forming PI3P in the omegasome membrane and recruiting downstream ubiquitin-like conjugation systems that convert LC3-I to LC3-II (4). Fully formed autophagosomes then fuse with lysosomes (autolysosomes), degrade the sequestered cargo *via* lysosomal hydrolases and recycle macromolecule components (5). Several drugs can interfere with the autophagic pathway by inhibiting or activating different parts of the process (see also **Table 1**). Drugs as rapamycin, resveratrol and nitazoxanide, that inhibit mTOR, or carbamazepine, metformin and pyrazinamide, that activate AMPK, induce autophagy. Bedaquiline, ambroxol and linezolid increase the formation of autophagosomes. Loperamide and valproic acid increase the colocalization of LC3-decorated autophagosomes with *M. tuberculosis*. Ibrutinib and isoniazid facilitate the fusion of phagosome and lysosome. Vitamin D3 (1,25D3) induces the expression of antimicrobial peptides as cathelicidin and upregulates the expression of Beclin 1 and ATG5, that are pivotal for the autophagosome formation. On the other hand, azithromycin was demonstrated to inhibit the acidification of the autolysosome impairing *M. abscessus* degradation.

Briefly, after pathogen recognition by host cells, the first step is the formation of the isolation membrane, which starts to grow and expand in size until sequestration and the surrounding of the target and finally closure to form the autophagosome. Subsequently, autophagosomes fuse with lysosomes to generate autolysosomes through elimination and recycling the sequestered charges *via* the lysosomal proteases (**Figure 1**) (28). A large number of proteins have been identified as highly relevant in different stages of control and action in autophagic flow. Several cell types have autophagy as an effector mechanism for homeostatic/immune functions as skin cells like keratinocytes and macrophages (**Figure 1**) (47).

A wide variety of signals regulates the activation of autophagy. The induction of autophagy can occur through the recognition of microbial factors that are ubiquitinated and recognized by autophagy cargo adaptor proteins (these include p62 (sequestosome 1), NBR1 (neighbor of BRCA1 gene 1 protein), NDP52 (calcium binding and coiled-coil domain 2), optineurin and galectin) or can occur by the production of reactive oxygen radicals and IFN-γ-mediated proteolysis, and autophagosome formation (43, 48–52). The autophagy pathway may be negatively regulated by PI3K (phosphoinositide 3-kinase)/Akt (protein kinase B)/mTOR (target of rapamycin in mammals) signalling (53). In contrast, the mitogen-activated protein kinase pathway (MAPK) can induce autophagy (54, 55).

## Autophagy as an Innate Immune Mechanism Against Mycobacterial Diseases

There is a strong relationship between autophagy signals and pattern recognition receptors, such as TLR (Toll-Like Receptors) that include TLR3, TLR4, TLR5, TLR6, TLR9, and the heterodimers TLR1/2, TLR7/8 that are capable of activating autophagy in macrophages, dendritic cells, and neutrophils (56–58). This activation occurs *via* signaling of the adaptor proteins MyD88 (myeloid differentiation factor 88) and TRIF (TIR-domain-containing adapter-inducing interferon-β). Xu and colleagues (59) demonstrated that after the stimulation of TLR4, positive LC3 (microtubule-associated protein 1A/1B-light chain 3) aggregates form in the macrophage cytoplasm and increase mycobacterial elimination through autophagy. Interestingly, for the LC3-aggregates induction, *via* TLR4 induction, it is necessary to activate the protein TRIF, as well as other proteins like RIP1 (receptor-interacting protein 1) and p38 for autophagic induction (56, 59). TLR4 acts as a pro-autophagic receptor in TRIF-dependent pathways. TLR4 induces the production of TNF (tumor necrosis factor) by a mechanism that is mediated both by reactive oxygen species (ROS) and nitrogen intermediates (i.e. nitric oxide), and by p38 and MAPK and the inhibition of these components may lead to total autophagy inactivity (60–62). Studies have shown that in LPS (lipopolysaccharide)-TLR4-mediated autophagy, activation of the transcription factor Nrf2 (nuclear factor erythroid 2–related factor 2) occurs, which leads to increased p62 transcription and formation of aggresome-like induced structures (ALIS) with subsequent autophagic degradation (63, 64), showing the ability of this receptor to link innate immunity with cellular oxidative response or adaptive immunity.

It is known that TLR receptors are of great importance for the activation of dendritic cells (DCs) and their subsequent maturation, some of these receptors such as TLR4 and TLR2 are already described as inducing an innate response against *M. tuberculosis* (65–67). Khan and colleagues (68) observed that the co-stimulus of CD40 and TLR4 leads to the production of pro-inflammatory cytokines such as IL-6, IL-12 and TNF, autophagy and death of mycobacteria. Interestingly, when they evaluated this co-stimulus as an adjunct to anti-TB therapy, they observed an increase *in vivo* and *in vitro* of the deadly potential of anti-TB drugs. Shin and colleagues (69) showed that stimulation of TLR2/1/CD14 by mycobacterial lipoprotein LpqH can activate antibacterial autophagy by activating vitamin D receptor signaling and inducing cathelicidin. They suggested that the TLR2/1/CD14-Ca^2+^-AMPK (Adenosine monophosphate-activated protein kinase)-p38 MAPK pathways contribute to cathelicidin-dependent expression, which played an important role in LpqH-induced autophagy. A study comparing the induction of autophagy by different species of mycobacteria found that non-pathogenic mycobacteria, such as *M. smegmatis*, induce a more robust autophagy response than *M. tuberculosis* (strain H37Rv) (70). The group observed a decrease in LC3-II protein expression when the TLR2 receptor was blocked, as well as a reduction in the colocalization of LC3 with *M. smegmatis* ΔpmmB (lipoglycan deficient mutant), suggesting the participation of TLR2 in the activation of autophagy during infection with *M. smegmatis* (70). *M. smegmatis* can also be recognized by NOD2 (nucleotide-binding oligomerization domain-containing protein 2) and dectin-2 receptors (71).

In addition to the TLR receptors, another group of innate receptors was the nucleotide-binding oligomerization domain (NLRs). It has already been described that the presence of the NOD2 receptor is capable of synergistically amplify the production of pro-inflammatory cytokines and their bactericidal activity (72). In previous studies, Khan and colleagues (73) have demonstrated that after the induction of both receptors, an increase in the bactericidal capacity of DCs *in vitro* was observed and they required a much lower dose of the drug to kill *M. tuberculosis*, in addition, activated DCs induced a more effective T cell response *in vivo* with an increase in autophagy (73, 74). Since pathogenic mycobacteria can modulate the autophagy machinery in skin cells, we hypothesize that autophagy may be a target for new therapeutic strategies against mycobacterial infections in the skin.

## Autophagy-Targeting Therapeutics Upon Mycobacterial Infection

Despite the efficacy of anti-TB treatment based on classic isoniazid and rifampicin, limitations in terms of drug resistance, duration of treatment, associated with the use of a complex treatment regimen (75), made the researchers use another strategy in the treatment of different bacterial disease. Besides, unlike infections caused by *M. tuberculosis* and *M. leprae* for which there is a well-established therapeutic regimen, there are no standardized and effective regimens for the treatment of non-tuberculosis mycobacteria (NTMs) (10). A promising strategy in the treatment of infectious diseases is the use of host-directed therapy. It works as an adjuvant therapy, which aims to enhance the main components of the host’s antimycobacterial effector mechanisms (76–79). Several studies on immunity, host-pathogen interactions, and host-directed interventions have shown that the antimycobacterial action of anti-TB drugs (standardized scheme) is associated with the induction of autophagy (40). Thus, several drugs used in the clinical area to treat infectious diseases may have their action through the autophagic process.

We previously showed that xenophagy is a crucial mechanism in the leprosy outcome. A functional autophagy pathway driven by IFN-γ and Beclin 1 in skin lesion macrophages was associated with the self-healing paucibacillary tuberculoid form of the disease, whereas a BCL2 (apoptosis regulator Bcl-2)-mediated block of Beclin 1 autophagy axis was linked to the progressive multibacillary lepromatous pole (35). While macrophages patrol the dermis, the human epidermis is enriched for Langerhans cells (LC). Langerhans cells restricted human immunodeficiency virus (HIV)-1 infection through the capture of viral particles by langerin and subsequent internalization into Birbeck granules and targeting of HIV-1 for destruction in the TRIM5 (tripartite motif-containing protein 5) auto lysosomal pathway (80), which in turn is induced by IFN-γ (81). In *M. leprae*-infected LC, the antimicrobial activity induced by IFN-γ treatment is achieved through autophagy, which improves the degradation of *M. leprae*-containing phagolysosomes and fine-tunes LC’s power to present antigens for T cells in a CD1a-restricted manner (82). Thus, IFN-γ therapy or a drug targeting autophagy on skin cells could be favorable to the clinical management of leprosy and other skin-related mycobacteriosis such as fish-tank granuloma (83) and Buruli ulcer (1), as well as outbreak associated postsurgical and tattoo ink infections caused by rapidly growing mycobacteria (2, 4). Indeed, the acid-fast bacilli clearance in the skin of multibacillary leprosy patients is accelerated when multidrug therapy is used along with an intradermal treatment with recombinant human IFN-γ (84).

Cell-based studies in leprosy have predominantly focused on dermal cells such as macrophages, neutrophils and T cells. In the dermis, macrophages are an important cell type that promote Th1-type responses, but there is evidence about the involvement of the epidermis in the development of reactional episodes (85) which are acute inflammatory episodes that can occur before, during or after the release of multidrug therapy, being responsible for the cases of disability caused by the disease (86). The relevance of autophagy as a drug target is not only restricted to the control of *M. leprae* infection but also to its potential to regulate the exacerbated inflammation associated with leprosy reactional episodes, as autophagy tempers inflammation by hijacking active inflammasomes for destruction (87). The downregulation of autophagy observed in skin lesion macrophages of multibacillary leprosy patients also predicts the reversal reaction onset. This impairment of the autophagic pathway correlates with the activation of NLRP3 (NALP3; NACHT, LRR and PYD domains-containing protein 3) inflammasome and IL-1β production, which drive the inflammatory status found in multibacillary patients when undergoing reversal reaction (36). On the other hand, due to Th2→Th1 shift and increased IFN-γ production, autophagy levels are restored in lepromatous patients when the reversal reaction episode is established, which in turn help to reduce the bacillary load in skin cells (35). Therefore, leprosy lesion skin cells can earn a dual benefit from the use of autophagy as a platform for drug development; both inflammasome and antimicrobial optimal activities can be reached by modulating autophagy to a certain level. However, some bacterial pathogens inhibit autophagosome maturation and promote bacterial replication, such as *M. tuberculosis* (88, 89). Given the background, Silva and colleagues (35) demonstrated that live but not dead *M. leprae* can inhibit the autophagic flux in macrophages, which indicates a requirement for an active mycobacterial ESX-1 secretion system.

The ESX-1 secretion system is also involved in the targeting of *M. marinum* by LC3; however, ubiquitination does not seem to be necessary for this process (83). *Legionella pneumophila* and *Coxiella burnetii* also developed strategies to explore or subvert autophagy (88). Kim and colleagues (42) demonstrated that *M. abscessus* (UC22 – rough variant) induces autophagy and inhibits autophagic flow in murine macrophages. Also as observed, the lipid components of the clinical isolate UC22, which is highly virulent, play a critical role in the formation of the autophagosome. These data suggest that virulent *M. abscessus* can survive and grow within autophagosomes, preventing autophagosome-lysosome fusion and clearance from cells (42). A study demonstrates the role of lactoferrin, an antimicrobial peptide, in the autophagy of macrophages infected with *M. avium*. D-lactoferrin inhibits intracellular growth of *M. avium* and, at the same time, leads to structural changes in infected macrophages leading to increased lysosomal content and increased numbers of autophagic vesicles (90).

P-aminosalicylate, one of oldest drugs used against tuberculosis, inhibits the assimilation of iron (91). Depletion of iron is strongly associated with increased expression and accumulation of regulated in DNA damage and development 1 (REDD1), which inhibits mTOR activation, decrease phosphorylation of Akt and TSC2 (tuberous sclerosis complex 2) (92, 93). Iron depletion was also shown to increase the activation of HIF-1α (hypoxia-inducible factor) and AMPK and induce autophagy (92, 94).

Zinc has been shown to be a positive regulator of autophagy in several different cell types and conditions, increasing the production of ROS, the formation and turnover of autophagosomes and cellular clearance (95–101). Nevertheless, zinc depletion was found to induce non-selective autophagy in yeast to release zinc recycled from zinc-rich proteins (91, 102, 103), demonstrating the key role of autophagy on zinc homeostasis. Zinc chelation was found to arrest autophagy and impair lysosomal acidification (95, 104). Phosphorylation of ERK1/2 is necessary for the regulation of zinc-induced autophagy by either activating the Beclin 1-PI3K complex or by promoting disassembly of mTOR complex but the mechanisms in which zinc modulates autophagy are still not completely understood (95, 99, 105). Uncoupling of autophagy and zinc homeostasis in the airway epithelial cells was demonstrated to be a fundamental mechanism in the pathogenesis of chronic obstructive pulmonary disease (106). In TB, previous studies have shown that zinc levels in the peripheral blood decrease with age and during active disease but are improved after the beginning of treatment with anti-TB drugs (107–111). Oral zinc supplementation in Brazilian children exposed to adults with pulmonary TB was demonstrated to increase the positivity of tuberculin test (PPD) and induration size, decreasing false negative results (112). It is postulated that zinc supplementation could correct asymptomatic zinc deficiencies, improve the effect of autophagy-mediated therapy in TB, as well as giving a booster to immunity (109, 111, 112). There are currently several studies associating autophagy and infection by bacteria, including studies showing the different strategies developed by bacteria to inhibit the host’s autophagic responses (113–117), as well as studies that show that the activation of autophagy by starvation or by treatment with rapamycin restricts bacterial growth and is capable of improving cell resistance to infection (39, 40, 118–120). The therapeutic benefit of pharmacological agents that can modulate autophagy must be considered since a diverse variety of pathogens using autophagic machinery has been described in their favor. It is primary to understand whether the pathogen exploits this pathway as a whole (systemically) or just part of components to increase its intracellular replication and/or survival. Besides, it is necessary to consider whether the drug will act on all autophagic pathways or only on a specific component, which may, or may not, be used to replicate for the pathogen. For example, intracellular *Brucella abortus* (*B. abortus*) survives by promoting the formation of vacuoles containing *B. abortus*, which requires the activity of the autophagy initiation proteins PIK3C3 (phosphatidylinositol 3-kinase catalytic subunit type 3), ULK1 (serine/threonine-protein kinase ULK1), ATG (autophagy-related protein) 14L (Barkor; Beclin 1-associated autophagy-related key regulator), and Beclin 1, but not the autophagy activity stretching proteins ATG16L1, ATG4B, ATG5, ATG7 and LC3-II (121). In this condition, the use of inhibitors of the autophagy protein conjugation systems or inhibitors of autophagosome maturation would not have a protective effect against the survival of this bacterium. Still in this context, it is important to consider those patients who are affected by infections (for example, TB) that can be eliminated if autophagy is regulated positively, but who are co-infected with pathogens that use the autophagic pathway in their favor, such as concomitant infections with the Hepatitis B virus and HIV (122). Under other conditions, the co-infected patient is favored by autophagic activation, as is the case of patients with cystic fibrosis (CF) who are treated with cysteamine. The autophagic stimulus mediated by cysteamine in macrophages of cystic fibrosis (with the CFTRdel506 mutation) patients favors the clearance of *Pseudomonas aeruginosa*, a bacterium that frequently infects the lungs of CF patients (123). Therefore, it is primary to understand the differences between each stimulus, pathogen, and the type of cell under study so that the use of this route as a target for the development of antimycobacterial drugs can be advanced.

## Treatments Inducing Autophagy During Tuberculous Mycobacterial Infection

When autophagy studies were started, the only drug that was able to chronically induce this pathway was rapamycin. There is evidence of its antimycobacterial activity, where it has been observed that it significantly inhibits infection by *M. kansasii*, *M. avium*, Bacillus Calmette–Guérin (BCG), and virulent strains of *M. tuberculosis* (124, 125). However, the adverse effects of rapamycin (which were not associated with autophagy induction) made this drug unattractive for use. Several drugs are capable of inducing autophagy and treating mycobacterial diseases, some examples are summarized in **Table 1** and their activities are illustrated in **Figure 1**.

**Table 1:** Therapeutic strategies of drug repositioning targeting autophagy of host cells against mycobacterial diseases.

Drugs	*Mycobacteria*	Model	Mechanism of Action	Reference
Rapamycin	*M. avium* subspecies *paratuberculosis* (MAP)	Inhibition of MAP growth *in vitro* (BACTEC radiometric 7H12 broth)	Inhibition of mTOR	Greenstein et al. (124)
Rapamycin	*M. smegmatis*	Murine bone marrow derived macrophages (BMDM) and RAW264.7 macrophages	Inhibition of mTOR	Zullo et al. (125)
Ambroxol	*M. tuberculosis*	BMDM and primary human macrophages	Increased autophagosomes production	Choi et al. (126)
Metformin*	*M. tuberculosis*	Monocytes differentiated to macrophage	Increases AMPK expression, inducing phosphorylation of ULK1	Singhal (127)
		(THP-1 cell line)		
Carbamazepine*	*M. tuberculosis*	Primary human macrophages	Lowers myoinositol levels, activates AMPK and induces autophagy in an mTOR independent manner	Cárdenas et al. (128); Schiebler et al. (129)
		Infection of C57BL/6		
		mice with MDR strain		
Valproic acid*	*M. tuberculosis*	Primary human macrophages	Increases colocalization of LC3 with Mtb	Schiebler et al. (129); Juárez et al. (130)
Loperamide	*M. tuberculosis*	Primary human macrophages	Decreases the production of TNF and increases the colocalization of LC3 with Mtb	Juárez et al. (130)
Bedaquiline*	*M. tuberculosis*	Human differentiated monocytes (U-937 cell line)	Increases the formation of autophagosomes	Genestet et al. (131)
Linezolid*	*M. tuberculosis*	Human differentiated monocytes (U-937 cell line)	Increases the formation of autophagosomes	Genestet et al. (131)
Resveratrol	*M. tuberculosis*	MIC values were determined against *M. tuberculosis* using the standard microbroth dilution method	Inhibits of mTOR	Sun et al. (132); Park et al. (133)
Baicalin	*M. tuberculosis*	RAW264.7 macrophages	Induces autophagy by inhibiting the PI3K/Akt/mTOR pathway	Zhang et al. (134)
Azithromycin*	*M. abscessus*	Primary human macrophages and C57BL/6 mice	Blocks lysosomal acidification	Renna et al. (135)
Rifabutin*	*M. abscessus*	MICs in dose-response assays were determined by the broth microdilution method	Undefined	Aziz et al. (136)
Nitazoxanide	*M. leprae*	C57BL/6 mice	mTOR inhibition by TSC2	Bailey et al. (137)
Isoniazid	*M. tuberculosis*	Primary BMDMs, human primary monocytes, and MDMs	Facilitates phagosome-lysosome fusion	Kim et al. (40)
Pyrazinamide	*M. tuberculosis*	Primary BMDMs, human primary monocytes, and MDMs	Activates AMPK and induces autophagy	Kim et al. (40)
Vitamin D3	*M. tuberculosis*	Human macrophages	Stimulation of VDR to induce cathelicidin expression; upregulation the expression of Atg5 and Beclin-1	Jo, (138); Palucci & Delogu, (139)
Vitamin D3	*M. leprae*	Peripheral monocytes	Stimulation of VDR to induce cathelicidin expression	Krutzik et al. (140), Montoya et al. (141)
Ibrutinib	*M. tuberculosis*	Monocytes differentiated to macrophage	Facilitates phagosome-lysosome fusion	Hu et al. (142)
		(THP-1 cell line) and C57BL/6 mice		
Iron	–	DN TfR-1 and DMT-1 CKO model	Iron depletion increases the activation of HIF-1α (hypoxia-inducible factor) and AMPK.	Wu et al. (94); Fretham et al. (92)
Verapamil	*M. tuberculosis*	BMDM from ATG5(flox/flox) (control) and ATG5(flox/flox) Lyz-Cre mice; Human monocytes	Inhibits Ca2^+^ channel, cytosolic	Abate et al. (143)
	*M. bovis* BCG		Ca2^+^↓	
Zinc	–	MCF-7 cells	Increasing the formation and turnover of autophagosomes	Hwang et al. (95);
		(human breast cancer cell line)		Cho et al. (104)
Simvastatin	*M. tuberculosis*	Peripheral blood mononuclear cells (PBMCs)	Increases the autophagic flux (autophagolysosomes)	Guerra-De-Blas et al. (144)
		PBMCs or MDMs from patients with familial hypercholesterolemia (FH) and C57BL/6 mice	Reduction of membrane cholesterol levels promotes phagosomal maturation (monocyte autophagy)	Parihar et al. (145)
Rosuvastatin	*M. tuberculosis*	PBMCs or MDMs from patients with familial hypercholesterolemia (FH) and C57BL/6 mice	Reduction of membrane cholesterol levels promotes phagosomal maturation (monocyte autophagy)	Parihar et al. (145)
Omadacycline	*Mycobacterium abscessus*	Broth microtiter dilution assay	–	Shoen et al. (146)
	*Mycobacterium chelonae*			
	*Mycobacterium fortuitum*			
Tigecycline	*Mycobacterium abscessus*	Broth microtiter dilution assay	–	Shoen et al. (146)
	*Mycobacterium chelonae*			
	*Mycobacterium fortuitum*			

*Repurposed Drugs.

Among the various drugs described in the literature with pro-autophagic properties, ambroxol (126), metformin (127), verapamil (143), carbamazepine (128, 129), valproic acid (129, 130), and loperamide (130) are already approved for clinical use in different pathologies. The strategy of using drugs with a known safety profile for new indications related to autophagy is attractive because they do not need to undergo a complete toxicological assessment (18, 147, 148).

Regarding the pro-autophagic property of ambroxol, it has been shown to potentiate the antimicrobial activity of rifampicin in the murine model in trials for TB (126). The antidiabetic drug metformin reduced the intracellular growth of *M. tuberculosis* in a manner dependent on AMPK. Also, metformin was able to induce reactive mitochondrial oxygen species and facilitate phagosome-lysosome fusion (127). However, a more recent study failed to show the improvement in the bacterial activity of antituberculosis drugs by metformin in the murine model (149). This data makes us reflect on the importance of considering whether the anti-TB drug may or may not alter the pharmacokinetics of the repositioning drug. The use of rifampicin in this more recent study (149) may have altered the pharmacokinetics of metformin. Besides, it is also prudent to pay attention to the differences in the experimental design carried out to assess the effectiveness of the therapy, which can be combined (149) or used alone (monotherapy) (127).

Initial studies that evaluated the effect of verapamil and its analogs on macrophages infected with *M. tuberculosis* showed that the structural analog KSV21 had an additive effect on the inhibitory antimicrobial activity of Isoniazid and Rifampicin (143). In addition, the antibiotics isoniazid and pyrazinamide, two first-line cocktail drugs used to treat TB, exert their antimycobacterial activity through autophagy (40).

Recently, the impact of linezolid and bedaquiline on the intra-macrophagic behavior of *M. tuberculosis* has been reported. It was observed that the anti-Mtb effect of these new drugs occurred *via* activation of autophagy and increased formation of autolysosomes in infected macrophages (131). Bedaquiline induces metabolic stress in *M. tuberculosis*, which results in the accumulation of NADH (nicotinamide adenine dinucleotide), followed by the generation of ROS (subsequently generating ROS by the bacteria) (150). Although not directly proven, ROS can trigger autophagy activation and be responsible for antibiotic-induced death of *M. tuberculosis* (151).

Resveratrol has also been studied for its antioxidant effect and its role in inducing autophagy. Regarding the antioxidant effect, resveratrol can increase the activity of antioxidant enzymes and works by eliminating free radicals (152, 153). Resveratrol has inhibitory activity on the mTOR molecule (133, 154). Other studies have shown antibacterial properties, mainly activity against gram-positive bacteria, flavonoid, and resveratrol (132). Still, on drugs capable of stimulating the autophagic death of *M. tuberculosis*, the anticonvulsant drug carbamazepine was able to induce autophagy in mice infected with the multidrug-resistant *M. tuberculosis* strain, resulting in a decrease in their bacterial load and improvement in pulmonary pathology (129). It was observed that carbamazepine induces antimicrobial autophagy due to decreased levels of Myoinositol (by blocking myoinositol uptake) into a pathway independent of mTOR. Furthermore, it was seen that this drug also activates AMPK (128). In that same study, the group described the induction of autophagy by the drug valproic acid, another anticonvulsant drug (129), which favored the increase in the co-localization of LC3 with *M. tuberculosis*, an effect similar to that observed after treatment with anti-diarrhea medication loperamide (130). Unlike carbamazepine, which activates AMPK, the induction of autophagy by baicalin in macrophages infected by *M. tuberculosis* occurred through inhibition of the PI3K/Akt/mTOR pathway. Additionally, baicalin showed a suppressive effect on the activation of the NLRP3 inflammasome *via* PI3K/Akt/NF-κB (nuclear factor-κB), as well as reduced the levels of the pro-inflammatory cytokine IL-1β (134). Both the induction of autophagy and the inhibition of NF-κB contribute to limit the activation of the NLRP3 inflammasome. Autophagy can limit the activation of the inflammasome indirectly or directly. Indirectly, it can reduce endogenous stimuli that favor the activation of the inflammasome (155, 156) and can directly inhibit the autophagic degradation of inflammasome components (87, 157).

Fluvastatin is a statin class drug currently used to treat hypercholesteromia and prevent cardiovascular disease, by blocking the enzyme hydroxy-methyl-glutaryl-CoA (HMG-CoA) reductase, which catalyzes a key step in cholesterol synthesis. Fluvastatin was demonstrated to be effective in targeting not only the mycobacteria but also increasing the ability of the host cells to eliminate *M. tuberculosis* infection (158). Other statins, including simvastatin and rosuvastatin were also demonstrated to control *M. tuberculosis* infection by promoting phagosomal maturation and autophagy (145).

Some studies demonstrated the protective role of autophagy in excessive inflammation during *M. tuberculosis* infection (159). Based on these studies, we conclude that autophagy plays an important role in the fight against TB, by direct killing of the pathogen, while also avoiding excessive inflammatory damage. This makes an antimycobacterial agent that has autophagy as a pharmacological target, a promising candidate to assist in therapy directed at the host.

## Role of Autophagy in Therapeutic Approaches for NTMs and Skin Diseases

The treatment of nontuberculous mycobacteriosis is not very rewarding. Currently, the proposed therapeutic regimen for infection with NTMs is based on the use of macrolides (clarithromycin or azithromycin), ethambutol, and rifamycins (160). Azithromycin is a potent antibiotic and is often prescribed for prophylaxis and treatment regimens of mycobacterial infections (10). However, one study reported that long-term use of azithromycin by adults with CF increased the risk of infection with *M. abscessus*. That was observed because the therapeutic dosage of azithromycin impaired autophagic degradation (135). That is, these data emphasize the importance of autophagy in the host’s response to infection by NTMs.

The challenge of treating lung diseases caused by *M. abscessus* is related to antibiotic resistance, including all first-line drugs for anti-TB treatment (161, 162). Even rifampicin, which has bactericidal activity against *M. tuberculosis* and *M. leprae*, has low potency against *M. abscessus*. Although rifampicin is part of the treatment regimens established for *M. kansasii* and *Mycobacterium avium* complex infections, it is not recommended against *M. abscessus* (163, 164). Recently, rifabutin (of the rifamycin group) was shown, through its bactericidal activity, to be effective against strains of clinical isolates from the three subspecies of the *M. abscessus* complex (subsp. *abscessus*, subsp. *massiliense*, and subsp. *bolletii*) (136). Recently, the *in vitro* activity of omadacycline and tigecycline against clinical isolates of *M. abscessus*, *M. chelonae* and *M. fortuitum* was evaluated (146). Omadacycline, a new tetracycline analog, approved for the treatment of acute bacterial skin and skin structure infections (ABSSSI) (165) showed activity against the three clinical isolates (146). There are reports that these microorganisms have been identified in postoperative infections caused by mycobacteria, including the three opportunistic pathogens: *M. fortuitum* (166), *M. abscessus* (167) and *M. chelonae* (168). Postoperative infections have been reported after orthopedic, laparoscopic, ophthalmic procedures and cosmetic operations (mainly liposuction, abdominoplasty, rhinoplasty) (169, 170). *M. chelona*e can cause localized skin infection after being accidentally inoculated from the environment (pedicure beds, water heaters, and tattoo parlors) (171, 172). In immunocompromised patients, the infection caused by this mycobacterium can manifest itself as a disseminated skin disease. A case report demonstrated *M. chelonae* skin and soft tissue infection in a patient with chronic lymphocytic leukemia (LLC) who was using ibrutinib, an oral drug, which acts by inhibiting Bruton tyrosine kinase (BTK) for the treatment of various malignant B-cell diseases (173, 174). After 6 months of therapy with ibrutinib, the 85-year-old man developed skin lesions on his arms and legs (175). Fiocari and colleagues (176) showed that ibrutinib promotes an M2 phenotype by modifying the function of macrophages/monocytes in the LLC. Taken together, these results showed that ibrutinib can have detrimental consequences on the microbicidal response in patients treated with ibrutinib. On the other hand, a more current study reported the impact of the drug ibrutinib on the intra-macrophagic behavior of *M. tuberculosis*. It was observed that the anti-TB effect of this medication occurred *via* activation of autophagy and facilitates phagosome-lysosome fusion in infected macrophages (142).

Nitazoxanide has also been studied for its role in inducing autophagy. The use of nitazoxanide in C57BL/6 mice infected with *M. leprae* showed a bactericidal action similar to that of rifampicin, an antibiotic used in the therapeutic regimen against leprosy (137). Based on this study, nitazoxanide (NTZ) may be an effective option for the treatment of leprosy (137).

The epidermis is composed mainly by keratinocytes, which contributes to the defense responses against various stimuli in the environment (177). Numerous findings indicate that autophagy plays an important role in the biology and pathology of keratinocytes (177). It has already been seen that calcipotriol, a vitamin D analog, has the ability to induce autophagy in keratinocytes (178). Analogous vitamin D molecules have been used to treat different skin diseases, such as psoriasis, lamellar ichthyosis and epidermolytic hyperkeratosis (179). The autophagic pathway converges with the vitamin D3-cathelicidin pathway, which is preferably seen in the paucibacillary form of leprosy (140, 141). Vitamin D3 induces autophagy *via* cathelicidin in macrophages infected with *M. tuberculosis*, with cathelicidin being required for IFNγ-mediated antimicrobial activity (180, 181). Also, 1,25(OH)2D3-induced LL-37 (C-terminal antimicrobial peptide) enhances the colocalization of mycobacterial phagosomes and autophagosomes (182). Vitamin D3 has been used successfully in the treatment of patients with TB (183). Vitamin D3 could be one of the components for the treatment of leprosy and other chronic infectious diseases in which the cellular immune response is unregulated (184, 185). Vitamin D prevents tissue damage through the negative regulation of perforin, granzyme B and granulisine in cytotoxic T lymphocytes (186).

Many species of mycobacteria that cause skin infections are considered to have a natural ability to acquire resistance to antibiotics and to have a significant reduction in sensitivity to antibiotics, which makes treatment efficacy more difficult by increasing failure rates (187, 188). Thus, using therapies directed at the host, such as those that induce autophagy, to inhibit bacterial cell release and form biofilms or bacterial media can increase the effectiveness of currently available antibiotics, i.e. azithromycin (135) and verapamil (143, 189) already mentioned in the text, as well as, Carvacrol (190–193), Tetracycline (146, 194, 195), Thioridazine (196–199) and, Mefloquine (200, 201).

## Conclusion

This review describes the potential of host cell autophagy as a target for the development of new strategies against mycobacterial diseases. There are few studies focusing on skin cell autophagy during mycobacterial infections but in this review we summarized autophagy mechanisms in some cells most relevant to skin mycobacterial diseases. In addition, drug repurposing presents itself as a promising perspective in the control of infections caused by mycobacteria, being used in isolation or complementary to existing treatments. Some challenges still need to be faced, such as the understanding of the mechanisms used by different species of mycobacteria to induce autophagy, the evaluation of host cell autophagy by different clinical isolates, including resistant strains, the impact of a therapy directed at the host cell in cases where there is co-infection and, finally, if the use of a drug in combination with current therapeutic regimens will have a beneficial effect on bacillary load.

## Author Contributions

TB, RP, BS, MG, and RP wrote the manuscript. TB, RP, and MG made the table and the figure. RP and MD provided intellectual output in the manuscript. All authors contributed to the article and approved the submitted version.

## Funding

We thank CAPES, FAPERJ, and CNPq funding institutions for all their financial support. This study was partially supported by the Coordination for the Improvement of Higher Education Personnel (Coordenação de Aperfeiçoamento de Pessoal de Nível Superior - CAPES) - Finance Code 001. National Council for Scientific and Technological Development (CNPq) - Finance Code 303834/2017-0. Rio de Janeiro Carlos Chagas Filho Research Foundation (FAPERJ) - Finance Code E-26/010.002231/2019.

## Conflict of Interest

The authors declare that the research was conducted in the absence of any commercial or financial relationships that could be construed as a potential conflict of interest.

## References

[1] Franco-Paredes C Marcos LA Henao-Martínez AF Rodríguez-Morales AJ Villamil-Gómez WE Gotuzzo E. Cutaneous Mycobacterial Infections. Clin Microbiol Rev (2018) 32(1):e00069–18. 10.1128/CMR.00069-18 PMC6302357 30429139

[2] Duarte RS Lourenço MCS Fonseca LDS Leão SC Amorim EDLT Rocha ILL. Epidemic of Postsurgical Infections Caused by Mycobacterium Massiliense. J Clin Microbiol (2009) 47(7):2149–55. 10.1128/JCM.00027-09 PMC2708466 19403765

[3] Khan FA Khakoo R. Nontuberculous Mycobacterial Cutaneous Infections: An Updated Review. Cutis (2011) 88(4):194–200. 22106729

[4] Kennedy BS Bedard B Younge M Tuttle D Ammerman E Ricci J. Outbreak of *Mycobacterium Chelonae* Infection Associated With Tattoo Ink. New Engl J Med (2012) 367(11):1020–4. 10.1056/nejmoa1205114 PMC10973765 22913660

[5] Gervais P Manuel O Jaton K Giulieri S. Skin Infections Due to Rapid-Growing Mycobacteria. Rev Med Suisse (2014) 10(427):931–4. 24843991

[6] Pinheiro RO Schmitz V Silva BJ de A Dias AA de Souza BJ. Innate Immune Responses in Leprosy. Front Immunol (2018) 9:518. 10.3389/fimmu.2018.00518 29643852 PMC5882777

[7] de Macedo CS Lara FA Pinheiro RO Schmitz V de Berrêdo-Pinho M Pereira GM. New Insights Into the Pathogenesis of Leprosy: Contribution of Subversion of Host Cell Metabolism to Bacterial Persistence, Disease Progression, and Transmission. F1000Research (2020) 9:F1000 Faculty Rev–70. 10.12688/f1000research.21383.1 PMC6996526 32051758

[8] Johansen MD Herrmann JL Kremer L. Non-Tuberculous Mycobacteria and the Rise of *Mycobacterium Abscessus* . Nat Rev Microbiol (2020) 18(7):392–407. 10.1038/s41579-020-0331-1 32086501

[9] Costa LL Veasey JV. Diagnosis of Cutaneous Tuberculosis (Lymph Node Scrofuloderma) Using the Xpert MTB/RIF® Method. Anais Bras Dermatol (2021) 96(1):82–4. 10.1016/j.abd.2020.01.009 PMC7838117 33279316

[10] Griffith DE Aksamit T Brown-Elliott BA Catanzaro A Daley C Gordin F. An Official ATS/IDSA Statement: Diagnosis, Treatment, and Prevention of Nontuberculous Mycobacterial Diseases. Am J Respir Crit Care Med (2007) 175(4):367–416. 10.1164/rccm.200604-571ST 17277290

[11] Forbes BY Hall GS Miller MB Novak S Rowlinson MC Salfinger M. Practice Guidelines for Clinical Microbiology Laboratories: Mycobacteria. Clin Microbiol Rev (2018) 31(2):e00038–17. 10.1128/CMR.00038-17 PMC5967691 29386234

[12] Wang SH Pancholi P. Mycobacterial Skin and Soft Tissue Infection. Curr Infect Dis Rep (2014) 16(11):438. 10.1007/s11908-014-0438-5 25339245

[13] Barbagallo J Tager P Ingleton R Hirsch RJ Weinberg JM. Cutaneous Tuberculosis: Diagnosis and Treatment. Am J Clin Dermatol (2002) 3(5):319–28. 10.2165/00128071-200203050-00004 12069638

[14] Zyl LV du Plessis J Vilijoen J. Cutaneous Tuberculosis Overview and Current Treatment Regimens. Tuberculosis (2015) 95(6):629–38. 10.1016/j.tube.2014.12.006 26616847

[15] Bravo FG Gotuzzo E. Cutaneous Tuberculosis. Clinics Dermatol (2007) 25(2):173–80. 10.1016/j.clindermatol.2006.05.005 17350496

[16] Rahman SA Singh Y Kohli S Ahmad J Ehtesham NZ Tyagi AK. Comparative Analyses of Nonpathogenic, Opportunistic, and Totally Pathogenic Mycobacteria Reveal Genomic and Biochemical Variabilities and Highlight the Survival Attributes of *Mycobacterium Tuberculosis* . mBio (2014) 5(6):e02020. 10.1128/mbio.02020-14 25370496 PMC4222108

[17] Carranza C Chavez-Galan L. Several Routes to the Same Destination: Inhibition of Phagosome-Lysosome Fusion by *Mycobacterium Tuberculosis* . Am J Med Sci (2019) 357(3):184–94. 10.1016/j.amjms.2018.12.003 30797501

[18] Kim YS Silwal P Kim SY Yoshimori T Jo EK. Autophagy-Activating Strategies to Promote Innate Defense Against Mycobacteria. Exp Mol Med (2019) 51(12):1–10. 10.1038/s12276-019-0290-7 PMC6906292 31827065

[19] Kim JK Silwal P Jo EK. Host-Pathogen Dialogues in Autophagy, Apoptosis, and Necrosis During Mycobacterial Infection. Immune Netw (2020) 20(5):e37. 10.4110/in.2020.20.e37 33163245 PMC7609165

[20] Chai Q Wang L Liu CH Ge B. New Insights Into the Evasion of Host Innate Immunity by *Mycobacterium Tuberculosis* . Cell Mol Immunol (2020) 17(9):901–13. 10.1038/s41423-020-0502-z PMC7608469 32728204

[21] Ankley L Thomas S Olive AJ. Fighting Persistence: How Chronic Infections With *Mycobacterium Tuberculosis* Evade T Cell-Mediated Clearance and New Strategies to Defeat Them. Infect Immun (2020) 88(7):e00916–19. 10.1128/IAI.00916-19 PMC7309621 32094248

[22] Bernard EM Fearns A Bussi C Santucci P Peddie CJ Lai RJ. *M. Tuberculosis* Infection of Human iPSC-derived Macrophages Reveals Complex Membrane Dynamics During Xenophagy Evasion. J Cell Sci (2020) 134(5):jcs252973. 10.1242/jcs.252973 32938685 PMC7710011

[23] Naeem MA Ahmad W Tyagi R Akram Q Younus M Liu X. Stealth Strategies of Mycobacterium Tuberculosis for Immune Evasion. Curr Issues Mol Biol (2021) 41:597–616. 10.21775/cimb.041.597 33068079

[24] Deter RL Baudhuin P De Duve C. Participation of Lysosomes in Cellular Autophagy Induced in Rat Liver by Glucagon. J Cell Biol (1967) 35(2):C11–6. 10.1083/jcb.35.2.c11 PMC2107130 6055998

[25] Klionsky DJ Emr SD. Autophagy as a Regulated Pathway of Cellular Degradation. Science (2000) 290(5497):1717–21. 10.1126/science.290.5497.1717 PMC2732363 11099404

[26] Wileman T. Autophagy as a Defence Against Intracellular Pathogens. Essays Biochem (2013) 55(1):153–63. 10.1042/BSE0550153 24070478

[27] Tsukamoto S Kuma A Mizushima N. The Role of Autophagy During the Oocyte-to-Embryo Transition. Autophagy (2008) 4(8):1076–8. 10.4161/auto.7065 18849666

[28] Gottlieb RA Carreira RS. Mitophagy as a Way of Life. Am J Physiol Cell Physiol (2010) 299(2):C203–210. 10.1152/ajpcell.00097.2010 PMC2928637 20357180

[29] Guan JL Simon AK Prescott M Menendez JA Liu F Wang F. Autophagy in Stem Cells. Autophagy (2013) 9(6):830–49. 10.4161/auto.24132 PMC3672294 23486312

[30] Deretic V Levine B. Autophagy Balances Inflammation in Innate Immunity. Autophagy (2018) 14(2):243–51. 10.1080/15548627.2017.1402992 PMC5902214 29165043

[31] Nakagawa I Amano A Mizushima N Yamamoto A Yamaguchi H Kamimoto T. Autophagy Defends Cells Against Invading Group A Streptococcus. Science (2004) 306(5698):1037–40. 10.1126/science.1103966 15528445

[32] Nakajima S Aikawa C Nozawa T Minowa-Nozawa A Toh H Nakagawa I. Bcl-Xl Affects Group a Streptococcus-Induced Autophagy Directly, by Inhibiting Fusion Between Autophagosomes and Lysosomes, and Indirectly, by Inhibiting Bacterial Internalization Via Interaction With Beclin 1-UVRAG. PloS One (2017) 12(1):e0170138. 10.1371/journal.pone.0170138 28085926 PMC5235370

[33] Muñoz-Sánchez S van der Vaart M Meijer AH. Autophagy and Lc3-Associated Phagocytosis in Zebrafish Models of Bacterial Infections. Cells (2020) 9(11):2372. 10.3390/cells9112372 PMC7694021 33138004

[34] Prajsnar TK Serba JJ Dekker BM Gibson JF Masud S Fleming A. The Autophagic Response to *Staphylococcus Aureus* Provides an Intracellular Niche in Neutrophils. Autophagy (2020) 17(4):888–902. 10.1080/15548627.2020.1739443 32174246 PMC8078660

[35] Silva BJ de A Barbosa MG deM Andrade PR Ferreira H Nery JA daC. Autophagy Is an Innate Mechanism Associated With Leprosy Polarization. PloS Pathog (2017a) 13(1):1–29. 10.1371/journal.ppat.1006103 PMC5215777 28056107

[36] de Mattos Barbosa MG de Andrade Silva BJ Assis TQ Prata RB da S Ferreira H. Autophagy Impairment is Associated With Increased Inflammasome Activation and Reversal Reaction Development in Multibacillary Leprosy. Front Immunol (2018) 9:1223. 10.3389/fimmu.2018.01223 29915584 PMC5994478

[37] Chen Z Shao X Wang C Hua Mh Wang Cn Wang X. Mycobacterium Marinum Infection in Zebrafish and Microglia Imitates the Early Stage of Tuberculous Meningitis. J Mol Neurosci (2018) 64(2):321–30. 10.1007/s12031-018-1026-1 29352446

[38] Zhang R Varela M Vallentgoed W Forn-Cuni G van der Vaart M Meijer AH. The Selective Autophagy Receptors Optineurin and p62 are Both Required for Zebrafish Host Resistance to Mycobacterial Infection. PloS Pathog (2019) 15(2):e1007329. 10.1371/journal.ppat.1007329 30818338 PMC6413957

[39] Gutierrez MG Master SS Singh SB Taylor GA Colombo MI Deretic V. Autophagy is a Defense Mechanism Inhibiting BCG and *Mycobacterium Tuberculosis* Survival in Infected Macrophages. Cell (2004) 119(6):753–66. 10.1016/j.cell.2004.11.038 15607973

[40] Kim JJ Lee HM Shin DM Kim W Yuk JM Jin HS. Host Cell Autophagy Activated by Antibiotics Is Required for Their Effective Antimycobacterial Drug Action. Cell Host Microbe (2012) 11(5):457–68. 10.1016/j.chom.2012.03.008 22607799

[41] Watson RO Bell SL MacDuff DA Kimmey JM Diner EJ Olivas J. The Cytosolic Sensor Cgas Detects *Mycobacterium Tuberculosis* DNA to Induce Type I Interferons and Activate Autophagy. Cell Host Microbe (2015) 17(6):811–9. 10.1016/j.chom.2015.05.004 PMC4466081 26048136

[42] Kim JK Lee HM Park KS Shin DM Kim TS Kim YS. MIR144* Inhibits Antimicrobial Responses Against *Mycobacterium Tuberculosis* in Human Monocytes and Macrophages by Targeting the Autophagy Protein DRAM2. Autophagy (2017b) 13(2):423–41. 10.1080/15548627.2016.1241922 PMC5324854 27764573

[43] Virgin HW Levine B. Autophagy Genes in Immunity. Nat Immunol (2009) 10(5):461–70. 10.1038/ni.1726 PMC2715365 19381141

[44] Levine B Mizushima N Virgin HW. Autophagy in Immunity and Inflammation. Nature (2011) 469(7330):323–35. 10.1038/nature09782 PMC3131688 21248839

[45] Ravenhill BJ Boyle KB von Muhlinen N Ellison CJ Masson GR Otten EG. The Cargo Receptor NDP52 Initiates Selective Autophagy by Recruiting the ULK Complex to Cytosol-Invading Bacteria. Mol Cell (2019) 74(2):320–329.e6. 10.1016/j.molcel.2019.01.041 30853402 PMC6477152

[46] Wen X Klionsky DJ. How Bacteria can Block Xenophagy: An Insight From *Salmonella* . Autophagy (2020) 16(2):193–4. 10.1080/15548627.2019.1666580 PMC6984612 31530078

[47] Puleston DJ Simon AK. Autophagy in the Immune System. Immunology (2013) 141(1):1–8. 10.1111/imm.12165 PMC3893844 23991647

[48] Wild P Farhan H McEwan DG Wagner S Rogov VV Brady NR. Phosphorylation of the Autophagy Receptor Optineurin Restricts Salmonella Growth. Science (2011) 333(6039):228–33. 10.1126/science.1205405 PMC3714538 21617041

[49] Oh JE Lee HK. Modulation of Pathogen Recognition by Autophagy. Front Immunol (2012) 3:44. 10.3389/fimmu.2012.00044 22566926 PMC3342359

[50] Paik S Jo EK. An Interplay Between Autophagy and Immunometabolism for Host Defense Against Mycobacterial Infection. Front Immunol (2020) 11:603951. 10.3389/fimmu.2020.603951 33262773 PMC7688515

[51] Randow F Youle RJ. Self and Nonself: How Autophagy Targets Mitochondria and Bacteria. in. Cell Host Microbe (2014) 15(4):403–11. 10.1016/j.chom.2014.03.012 PMC4238923 24721569

[52] Da Silva Prata RB de Mattos Barbosa MG de Andrade Silva BJ Oliveira JAP Bittencourt TL. Macrophages in the Pathogenesis of Leprosy. In: Macrophage activation - Biology and Disease. United Kingdom: Intech Open (2019), p. 1–19. 10.5772/INTECHOPEN.88754 IntechOpen.

[53] Heras-Sandoval D Pérez-Rojas JM Hernández-Damián J Pedraza-Chaverri J. The Role of PI3K/AKT/mTOR Pathway in the Modulation of Autophagy and the Clearance of Protein Aggregates in Neurodegeneration. Cell Signal (2014) 26(12):2694–701. 10.1016/j.cellsig.2014.08.019 25173700

[54] Krishna M Narang H. The Complexity of Mitogen-Activated Protein Kinases (Mapks) Made Simple. Cell Mol Life Sci (2008) 65(22):3525–44. 10.1007/s00018-008-8170-7 PMC11131782 18668205

[55] Zhou YY Li Y Jiang WQ Zhou LF. MAPK/JNK Signalling: A Potential Autophagy Regulation Pathway. Biosci Rep (2015) 35(3):1–10. 10.1042/BSR20140141 PMC4613668 26182361

[56] Delgado MA Elmaoued RA Davis AS Kyei G Deretic V. Toll-Like Receptors Control Autophagy. EMBO J (2008) 27(7):1110–21. 10.1038/emboj.2008.31 PMC2323261 18337753

[57] Fitzgerald KA Kagan JC. Toll-Like Receptors and the Control of Immunity. Cell (2020) 180(6):1044–66. 10.1016/j.cell.2020.02.041 PMC9358771 32164908

[58] Pellegrini JM Sabbione F Morelli MP Tateosian NL Castello FA Amiano NO. Neutrophil Autophagy During Human Active Tuberculosis is Modulated by SLAMF1. Autophagy (2020) 16:1–10. 10.1080/15548627.2020.1825273 32954947 PMC8496709

[59] Xu Y Jagannath C Liu X Sharafkhaneh A Kolodziejska KE Eissa NT. Toll-Like Receptor 4 Is a Sensor for Autophagy Associated With Innate Immunity. Immunity (2007) 27(1):135–44. 10.1016/j.immuni.2007.05.022 PMC2680670 17658277

[60] Asehnoune K Strassheim D Mitra S Kim JY Abraham E. Involvement of Reactive Oxygen Species in Toll-Like Receptor 4-Dependent Activation of NF-κb. J Immunol (2004) 172(4):2522–9. 10.4049/jimmunol.172.4.2522 14764725

[61] Zhou M Xu W Wang J Yan J Shi Y Zhang C. Boosting mTOR-dependent Autophagy Via Upstream TLR4-MyD88-MAPK Signalling and Downstream NF-κb Pathway Quenches Intestinal Inflammation and Oxidative Stress Injury. EBioMedicine (2018) 35:345–60. 10.1016/j.ebiom.2018.08.035 PMC6161481 30170968

[62] Hasan A Akhter N Al-Roub A Thomas R Kochumon S Wilson A. Tnf-α in Combination With Palmitate Enhances IL-8 Production Via the MyD88- Independent TLR4 Signaling Pathway: Potential Relevance to Metabolic Inflammation. Int J Mol Sci (2019) 20(17):4112. 10.3390/ijms20174112 PMC6747275 31443599

[63] Fujita KI Srinivasula SM. TLR4-Mediated Autophagy in Macrophages is a p62-dependent Type of Selective Autophagy of Aggresome-Like Induced Structures (ALIS). Autophagy (2011a) 7(5):552–4. 10.4161/auto.7.5.15101 PMC3127216 21412052

[64] Fujita KI Maeda D Xiao Q Srinivasula SM. Nrf2-mediated Induction of p62 Controls Toll-like receptor-4-driven Aggresome-Like Induced Structure Formation and Autophagic Degradation. Proc Natl Acad Sci USA (2011b) 108(4):1427–32. 10.1073/pnas.1014156108 PMC3029726 21220332

[65] Shinya E Owaki A Norose Y Sato S Takahashi H. Quick Method of Multimeric Protein Production for Biologically Active Substances Such as Human GM-CSF (Hgm-CSF). Biochem Biophys Res Commun (2009) 386(1):40–4. 10.1016/j.bbrc.2009.05.125 19497303

[66] Pompei L Jang S Zamlynny B Ravikumar S McBride A Hickman SP. Disparity in IL-12 Release in Dendritic Cells and Macrophages in Response to *Mycobacterium Tuberculosis* is Due to Use of Distinct TLRs. J Immunol (2007) 178(8):5192–9. 10.4049/jimmunol.178.8.5192 17404302

[67] Drage MG Pecora ND Hise AG Febbraio M Silverstein RL Golenbock DT. TLR2 and its Co-Receptors Determine Responses of Macrophages and Dendritic Cells to Lipoproteins of Mycobacterium Tuberculosis. Cell Immunol (2009) 258(1):29–37. 10.1016/j.cellimm.2009.03.008 19362712 PMC2730726

[68] Khan N Pahari S Vidyarthi A Aqdas M Agrewala JN. Stimulation Through CD40 and TLR-4 is an Effective Host Directed Therapy Against *Mycobacterium Tuberculosis* . Front Immunol (2016a) 7:386. 10.3389/fimmu.2016.00386 27729911 PMC5037235

[69] Shin DM Yuk JM Lee HM Lee SH Son JW Harding CV. Mycobacterial Lipoprotein Activates Autophagy Via TLR2/1/CD14 and a Functional Vitamin D Receptor Signalling. Cell Microbiol (2010) 12(11):1648–65. 10.1111/j.1462-5822.2010.01497 PMC2970753 20560977

[70] Bah A Lacarrière C Vergne I. Autophagy-Related Proteins Target Ubiquitin-Free Mycobacterial Compartment to Promote Killing in Macrophages. Front Cell Infect Microbiol (2016) 6:53. 10.3389/fcimb.2016.00053 27242971 PMC4863073

[71] Travassos LH Carneiro LAM Ramjeet M Hussey S Kim YG Magalhes JG. Nod1 and Nod2 Direct Autophagy by Recruiting ATG16L1 to the Plasma Membrane At the Site of Bacterial Entry. Nat Immunol (2010) 11(1):55–62. 10.1038/ni.1823 19898471

[72] Ferwerda G Girardin SE Kullberg BJ Le Bourhis L De Jong DJ Langenberg DML. NOD2 and Toll-Like Receptors are Nonredundant Recognition Systems of Mycobacterium Tuberculosis. PloS Pathog (2005) 1(3):279–85. 10.1371/journal.ppat.0010034 PMC1291354 16322770

[73] Khan N Pahari S Vidyarthi A Aqdas M Agrewala JN. NOD-2 and TLR-4 Signaling Reinforces the Efficacy of Dendritic Cells and Reduces the Dose of TB Drugs Against Mycobacterium Tuberculosis. J Innate Immun (2016b) 8(3):228–42. 10.1159/000439591 PMC6738777 26613532

[74] Khan N Vidyarthi A Pahari S Negi S Aqdas M Nadeem S. Signaling Through NOD-2 and TLR-4 Bolsters the T Cell Priming Capability of Dendritic Cells by Inducing Autophagy. Sci Rep (2016c) 6:19084. 10.1038/srep19084 26754352 PMC4709561

[75] Zumla A Rao M Parida SK Keshavjee S Cassell G Wallis R. Inflammation and Tuberculosis: Host-directed Therapies. in. J Internal Med (2015) 277(4):373–87. 10.1111/joim.12256 24717092

[76] Hawn TR Matheson AI Maley SN Vandal O. Host-Directed Therapeutics for Tuberculosis: can We Harness the Host? Microbiol Mol Biol Rev (2013) 77(4):608–27. 10.1128/mmbr.00032-13 PMC3973381 24296574

[77] Zumla A Rao M Wallis RS Kaufmann SHE Rustomjee R Mwaba P. Host-directed Therapies for Infectious Diseases: Current Status, Recent Progress, and Future Prospects. Lancet Infect Dis (2016) 16(4):e47–63. 10.1016/S1473-3099(16)00078-5 PMC7164794 27036359

[78] Machelart A Song OR Hoffmann E Brodin P. Host-Directed Therapies Offer Novel Opportunities for the Fight Against Tuberculosis. Drug Discovery Today (2017) 22(8):1250–7. 10.1016/j.drudis.2017.05.005 28533187

[79] Yang CS. Advancing Host-Directed Therapy for Tuberculosis. Microb Cell (2017) 4(3):105–7. 10.15698/mic2017.03.565 PMC5349197 28357397

[80] Ribeiro CMS Sarrami-Forooshani R Setiawan LC Zijlstra-Willems EM Van Hamme JL Tigchelaar W. Receptor Usage Dictates HIV-1 Restriction by Human TRIM5α in Dendritic Cell Subsets. Nature (2016) 540(7633):448–52. 10.1038/nature20567 27919079

[81] Kimura T Jain A Choi SW Mandell MA Schroder K Johansen T. TRIM-Mediated Precision Autophagy Targets Cytoplasmic Regulators of Innate Immunity. J Cell Biol (2015) 210(6):973–89. 10.1083/jcb.201503023 PMC4576868 26347139

[82] Dang AT Teles RMB Liu PT Choi A Legaspi A Sarno EN. Autophagy Links Antimicrobial Activity With Antigen Presentation in Langerhans Cells. JCI Insight (2019) 4(8):e126955. 10.1172/jci.insight.126955 PMC6538337 30996142

[83] Lerena MC Colombo MI. *Mycobacterium Marinum* Induces a Marked LC3 Recruitment to its Containing Phagosome That Depends on a Functional ESX-1 Secretion System. Cell Microbiol (2011) 13(6):814–35. 10.1111/j.1462-5822.2011.01581.x 21447143

[84] Sampaio E Malta A Sarno E Kaplan G. Effect of rhuIFN-gamma Treatment in Multibacillary Leprosy Patients. Int J Lepr Other Mycobact Dis (1996) 64(3):268–73. 8862260

[85] Cogen AL Walker SL Roberts CH Hagge DA Neupane KD Khadge S. Human Beta-Defensin 3 Is Up-Regulated in Cutaneous Leprosy Type 1 Reactions. PloS Neglect Trop Dis (2012) 6(11):e1869. 10.1371/journal.pntd.0001869 PMC3486878 23133681

[86] Scollard DM Adams LB Gillis TP Krahenbuhl JL Truman RW Williams DL. The Continuing Challenges of Leprosy. Clin Microbiol Rev (2006) 19(2):338–81. 10.1128/CMR.19.2.338-381.2006 PMC1471987 16614253

[87] Shi CS Shenderov K Huang NN Kabat J Abu-Asab M Fitzgerald KA. Activation of Autophagy by Inflammatory Signals Limits IL-1β Production by Targeting Ubiquitinated Inflammasomes for Destruction. Nat Immunol (2012) 13(3):255–63. 10.1038/ni.2215 PMC4116819 22286270

[88] Campoy E Colombo MI. Autophagy Subversion by Bacteria. Curr Topics Microbiol Immunol (2009) 335:227–50. 10.1007/978-3-642-00302-8_11 19802568

[89] Cemma M Brumell JHH. Interactions of Pathogenic Bacteria With Autophagy Systems. Curr Biol (2012) 22(13):R540–5. 10.1016/j.cub.2012.06.001 22790007

[90] Silva T Moreira AC Nazmi K Moniz T Vale N Rangel M. Lactoferricin Peptides Increase Macrophages’ Capacity to Kill *Mycobacterium Avium* . mSphere (2017b) 2(4):301–17. 10.1128/mSphere PMC5577653 28875176

[91] Periyasamy KM Ranganathan UD Tripathy SP Bethunaickan R. Vitamin D – A Host Directed Autophagy Mediated Therapy for Tuberculosis. Mol Immunol (2020) 127:238–44. 10.1016/j.molimm.2020.08.007 33039674

[92] Fretham SJB Carlson ES Georgieff MK. Neuronal-Specific Iron Deficiency Dysregulates Mammalian Target of Rapamycin Signaling During Hippocampal Development in Nonanemic Genetic Mouse Models. J Nutr (2013) 143(3):260–6. 10.3945/jn.112.168617 PMC3713018 23303869

[93] Watson A Lipina C McArdle HJ Taylor PM Hundal HS. Iron Depletion Suppresses mTORC1-directed Signalling in Intestinal Caco-2 Cells Via Induction of REDD1. Cell Signal (2016) 28(5):412–24. 10.1016/j.cellsig.2016.01.014 PMC4804389 26827808

[94] Wu Y Li X Xie W Jankovic J Le W Pan T. Neuroprotection of Deferoxamine on Rotenone-Induced Injury Via Accumulation of HIF-1 Alpha and Induction of Autophagy in SH-SY5Y Cells. Neurochem Int (2010) 57(3):198–205. 10.1016/j.neuint.2010.05.008 20546814

[95] Hwang JJ Ha NK Kim J Cho D Mi JK Kim Y. Zinc (II) Ion Mediates Tamoxifen-Induced Autophagy and Cell Death in MCF-7 Breast Cancer Cell Line. Biometals (2010) 23(6):997–1013. 10.1007/s10534-010-9346-9 20524045

[96] Lee S Koh J. Roles of Zinc and Metallothionein-3 in Oxidative Stress-Induced Lysosomal Dysfunction, Cell Death, and Autophagy in Neurons and Astrocytes. Mol Brain (2010) 3(1):30. 10.1186/1756-6606-3-30 20974010 PMC2988061

[97] Kim KW Speirs CK Jung DK Lu B. The Zinc Ionophore PCI-5002 Radiosensitizes Non-Small Cell Lung Cancer Cells by Enhancing Autophagic Cell Death. J Thorac Oncol (2011) 6(9):1542–52. 10.1097/JTO.0b013e3182208fac 21642866

[98] Hung H Huang W Pan C. Dopamine-and Zinc-Induced Autophagosome Formation Facilitates PC12 Cell Survival. Cell Biol Toxicol (2013) 29(6):415–29. 10.1007/s10565-013-9261-2 24077806

[99] Liuzzi JP Yoo C. Role of Zinc in the Regulation of Autophagy During Ethanol Exposure in Human Hepatoma Cells. Biol Trace Element Res (2013) 156(1-3):350–6. 10.1007/s12011-013-9816-3 24061963

[100] Pan R Timmins GS Liu W Liu KJ. Autophagy Mediates Astrocyte Death During Zinc-Potentiated Ischemia–Reperfusion Injury. Biol Trace Element Res (2015) 166(1):89–95. 10.1007/s12011-015-0287-6 PMC4470843 25758719

[101] Popp L Segatori L. Zinc Oxide Particles Induce Activation of the Lysosome–Autophagy System. ACS Omega (2019) 4(1):573–81. 10.1021/acsomega.8b01497

[102] Kawamata T Horie T Matsunami M Sasaki M Ohsumi Y. Zinc Starvation Induces Autophagy in Yeast. J Biol Chem (2017) 292(20):8520–30. 10.1074/jbc.M116.762948 PMC5437255 28264932

[103] Ding B Zhong Q. Zinc Deficiency: An Unexpected Trigger for Autophagy. J Biol Chem (2017) 292(20):8531–2. 10.1074/jbc.H116.762948 PMC5437256 28526691

[104] Cho YH Lee SH Lee SJ Kim HN Koh JY. A Role of Metallothionein-3 in Radiation-Induced Autophagy in Glioma Cells. Sci Rep (2020) 10(1):2015. 10.1038/s41598-020-58237-7 32029749 PMC7005189

[105] Liuzzi JP Guo L Yoo C Stewart TS. Zinc and Autophagy. Biometals (2014) 27(6):1087–96. 10.1007/s10534-014-9773-0 PMC4224969 25012760

[106] Roscioli E Tran HB Jersmann H Nguyen PT Hopkins E Lester S. The Uncoupling of Autophagy and Zinc Homeostasis in Airway Epithelial Cells as a Fundamental Contributor to COPD. Am J Physiol Lung Cell Mol Physiol (2017) 313(3):L453–65. 10.1152/ajplung.00083.2017 28596293

[107] Taneja DP. Observations on Serum Zinc in Patients of Pulmonary Tuberculosis. J Indian Med Assoc (1990) 88(10):280–1. 2090683

[108] Ray M Kumar L Prasad R. Plasma Zinc Status in Indian Childhood Tuberculosis: Impact of Antituberculosis Therapy. Int J Tuberculosis Lung Dis (1998) 2(9):719–25. 9755925

[109] Karyadi E Schultink W Nelwan RH Gross R Amin Z Dolmans WM. Poor Micronutrient Status of Active Pulmonary Tuberculosis Patients in Indonesia. J Nutr (2000) 130(12):2953–8. 10.1093/jn/130.12.2953 11110853

[110] Koyanagi A Kuffó D Gresely L Shenkin A Cuevas LE. Relationships Between Serum Concentrations of C-reactive Protein and Micronutrients, in Patients With Tuberculosis. Ann Trop Med Parasitol (2004) 98(4):391–9. 10.1179/000349804225003424 15228720

[111] Ghulam H Kadri SM Manzoor A Waseem Q Aatif MS Khan GQ. Status of Zinc in Pulmonary Tuberculosis. J Infect Dev Ctries (2009) 3(5):365–8. 10.3855/jidc.244 19759506

[112] Cuevas LE Almeida LM Mazunder P Paixão AC Silva AM Maciel L. Effect of Zinc on the Tuberculin Response of Children Exposed to Adults With Smear-Positive Tuberculosis. Ann Trop Paediatr (2002) 22(4):313–9. 10.1179/027249302125001967 12530280

[113] Yoshikawa Y Ogawa M Hain T Chakraborty T Sasakawa C. Listeria Monocytogenes ActA is a Key Player in Evading Autophagic Recognition. Autophagy (2009) 5(8):1220–1. 10.4161/auto.5.8.10177 19855178

[114] Shahnazari S Namolovan A Mogridge J Kim PK Brumell JH. Bacterial Toxins can Inhibit Host Cell Autophagy Through cAMP Generation. Autophagy (2011) 7(9):957–65. 10.4161/auto.7.9.16435 21606683

[115] Tattoli I Sorbara MT Philpott DJ Girardin SE. Bacterial Autophagy: The Trigger, the Target and the Timing. Autophagy (2012) 8(12):1848–50. 10.4161/auto.21863 PMC3541301 22932645

[116] Dong N. Structurally Distinct Bacterial TBC-like Gaps Link Arf Gtpase to Rab1 Inactivation to Counteract Host Defenses. Cell (2012) 150(5):1029–41. 10.1016/j.cell.2012.06.050 22939626

[117] O’Keeffe KM Wilk MM Leech JM Murphy AG Laabei M Monk IR. Manipulation of Autophagy in Phagocytes Facilitates Staphylococcus Aureus Bloodstream Infection. Infect Immun (2015) 83(9):3445–57. 10.1128/IAI.00358-15 PMC4534639 26099586

[118] Lapaquette P Bringer MA Darfeuille-Michaud A. Defects in Autophagy Favour Adherent-Invasive Escherichia Coli Persistence Within Macrophages Leading to Increased Pro-Inflammatory Response. Cell Microbiol (2012) 14(6):791–807. 10.1111/j.1462-5822.2012.01768.x 22309232

[119] Kuo SY Castoreno AB Aldrich LN Lassen KG Goel G Dančík V. Small-molecule Enhancers of Autophagy Modulate Cellular Disease Phenotypes Suggested by Human Genetics. Proc Natl Acad Sci USA (2015) 112(31):E4281–7. 10.1073/pnas.1512289112 PMC4534235 26195741

[120] Miao Y Li G Zhang X Xu H Abraham SN. A TRP Channel Senses Lysosome Neutralization by Pathogens to Trigger Their Expulsion. Cell (2015) 161(6):1306–19. 10.1016/j.cell.2015.05.009 PMC4458218 26027738

[121] Starr T Child R Wehrly TD Hansen B Hwang S López-Otin C. Selective Subversion of Autophagy Complexes Facilitates Completion of the Brucella Intracellular Cycle. Cell Host Microbe (2012) 11(1):33–45. 10.1016/j.chom.2011.12.002 22264511 PMC3266535

[122] Li J Liu Y Wang Z Liu K Wang Y Liu J. Subversion of Cellular Autophagy Machinery by Hepatitis B Virus for Viral Envelopment. J Virol (2011) 85(13):6319–33. 10.1128/JVI.02627-10 PMC3126540 21507968

[123] Ferrari E Monzani R Villella VR Esposito S Saluzzo F Rossin F. Cysteamine Re-Establishes the Clearance of Pseudomonas Aeruginosa by Macrophages Bearing the Cystic Fibrosis-Relevant F508del-CFTR Mutation. Cell Death Dis (2017) 8(1):e2544. 10.1038/cddis.2016.476 28079883 PMC5386380

[124] Greenstein RJ Su L Juste RA Brown ST. On the Action of Cyclosporine A, Rapamycin and Tacrolimus on M. Avium Including Subspecies Paratuberculosis. PloS One (2008) 3(6):e2496. 10.1371/journal.pone.0002496 18575598 PMC2427180

[125] Zullo AJ Jurcic Smith KL Lee S. Mammalian Target of Rapamycin Inhibition and Mycobacterial Survival are Uncoupled in Murine Macrophages. BMC Biochem (2014) 15(1):4. 10.1186/1471-2091-15-4 24528777 PMC3937017

[126] Choi SW Gu Y Peters RS Salgame P Ellner JJ Timmins GS. Ambroxol Induces Autophagy and Potentiates Rifampin Antimycobacterial Activity. Antimicrobial Agents Chemother (2018) 62(9):e01019–18. 10.1128/aac.01019-18 PMC6125555 30012752

[127] Singhal A Jie L Kumar P Hong GS Leow MKS Paleja B. Metformin as Adjunct Antituberculosis Therapy. Sci Trans Med (2014) 6(263):263ra159. 10.1126/scitranslmed.3009885 25411472

[128] Cárdenas-Rodríguez N Coballase-Urrutia E Rivera-Espinosa L Romero-Toledo A Sampieri AI Ortega-Cuellar D. Modulation of Antioxidant Enzymatic Activities by Certain Antiepileptic Drugs (Valproic Acid, Oxcarbazepine, and Topiramate): Evidence in Humans and Experimental Models. Oxid Med Cell Longevity (2013) 2013:598493. 10.1155/2013/598493 PMC3877618 24454986

[129] Schiebler M Brown K Hegyi K Newton SM Renna M Hepburn L. Functional Drug Screening Reveals Anticonvulsants as Enhancers of mTOR-Independent Autophagic Killing of *Mycobacterium Tuberculosis* Through Inositol Depletion. EMBO Mol Med (2015) 7(2):127–39. 10.15252/emmm.201404137 PMC4328644 25535254

[130] Juárez E Carranza C Sánchez G González M Chávez J Sarabia C. Loperamide Restricts Intracellular Growth of *Mycobacterium Tuberculosis* in Lung Macrophages. Am J Respir Cell Mol Biol (2016) 55(6):837–47. 10.1165/rcmb.2015-0383OC 27468130

[131] Genestet C Bernard-Barret F Hodille E Ginevra C Ader F Goutelle S. Antituberculous Drugs Modulate Bacterial Phagolysosome Avoidance and Autophagy in *Mycobacterium Tuberculosis*-Infected Macrophages. Tuberculosis (2018) 111:67–70. 10.1016/j.tube.2018.05.014 30029917

[132] Sun D Hurdle JG Lee R Lee R Cushman M Pezzuto JM. Evaluation of Flavonoid and Resveratrol Chemical Libraries Reveals Abyssinone II as a Promising Antibacterial Lead. ChemMedChem (2012) 7(9):1541–5. 10.1002/cmdc.201200253 PMC3516920 22847956

[133] Park D Jeong H Lee MN Koh A Kwon O Yang YR. Resveratrol Induces Autophagy by Directly Inhibiting mTOR Through ATP Competition Dohyun Park1. Sci Rep (2016) 23:6–21772. 10.1038/srep21772 PMC4763238 26902888

[134] Zhang Q Sun J Wang Y He W Wang L Zheng Y. Antimycobacterial and Anti-Inflammatory Mechanisms of Baicalin Via Induced Autophagy in Macrophages Infected With *Mycobacterium Tuberculosis* . Front Microbiol (2017) 8:2142. 10.3389/fmicb.2017.02142 29163427 PMC5673628

[135] Renna M Schaffner C Brown K Shang S Tamayo MH Hegyi K. Azithromycin Blocks Autophagy and may Predispose Cystic Fibrosis Patients to Mycobacterial Infection. J Clin Invest (2011) 121(9):3554–63. 10.1172/JCI46095 PMC3163956 21804191

[136] Aziz DB Low JL Wu ML Gengenbacher M Teo JWP Dartois V. Rifabutin Is Active Against *Mycobacterium Abscessus* Complex. Antimicrobial Agents Chemother (2017) 61(6):e00155–17. 10.1128/AAC.00155-17 PMC5444174 28396540

[137] Bailey MA Na H Duthie MS Gillis TP Lahiri R Parish T. Nitazoxanide is Active Against *Mycobacterium Leprae* . PloS One (2017) 12(8):e0184107. 10.1371/journal.pone.0184107 28850614 PMC5574600

[138] Jo EK. Innate Immunity to Mycobacteria: Vitamin D and Autophagy. Cell Microbiol (2010) 12(8):1026–35. 10.1111/j.1462-5822.2010.01491.x 20557314

[139] Palucci I Delogu G. Host Directed Therapies for Tuberculosis: Futures Strategies for an Ancient Disease. Chemotherapy (2018) 63(3):172–80. 10.1159/000490478 30032143

[140] Krutzik SR Hewison M Liu PT Robles JA Stenger S Adams JS. Il-15 Links Tlr2/1-Induced Macrophage Differentiation to the Vitamin D-Dependent Antimicrobial Pathway. J Immunol (2008) 181(10):7115–20. 10.4049/jimmunol.181.10.7115 PMC2678236 18981132

[141] Montoya D Cruz D Teles RMB Lee DJ Ochoa MT Krutzik SR. Divergence of Macrophage Phagocytic and Antimicrobial Programs in Leprosy. Cell Host Microbe (2009) 6(4):343–53. 10.1016/j.chom.2009.09.002 PMC2764558 19837374

[142] Hu Y Wen Z Liu S Cai Y Guo J Xu Y. Ibrutinib Suppresses Intracellular Mycobacterium Tuberculosis Growth by Inducing Macrophage Autophagy. J Infect (2020) 80(6):e19–26. 10.1016/j.jinf.2020.03.003 32171871

[143] Abate G Ruminiski PG Kumar M Singh K Hamzabegovic F Hoft DF. New Verapamil Analogs Inhibit Intracellular Mycobacteria Without Affecting the Functions of Mycobacterium-Specific T Cells. Antimicrobial Agents Chemother (2016) 60(3):1216–25. 10.1128/AAC.01567-15 PMC4775996 26643325

[144] Guerra-De-Blas PDC Bobadilla-Del-Valle M Sada-Ovalle I Estrada-Garcia I Torres-Gonzalez P Lopez-Saavedra A. Simvastatin Enhances the Immune Response Against Mycobacterium Tuberculosis. Front Microbiol (2019) 10:2097. 10.3389/fmicb.2019.02097 102097. 31616387 PMC6764081

[145] Parihar SP Guler R Khutlang R Lang DM Hurdayal R Mhlanga MM. Statin Therapy Reduces the Mycobacterium Tuberculosis Burden in Human Macrophages and in Mice by Enhancing Autophagy and Phagosome Maturation. J Infect Dis (2014) 209(5):754–63. 10.1093/infdis/jit550 24133190

[146] Shoen C Benaroch D Sklaney M Cynamon M. In Vitro Activities of Omadacycline Against Rapidly Growing Mycobacteria. Antimicrobial Agents Chemother (2019) 63(5):e02522–18. 10.1128/AAC.02522-18 PMC6496053 30858221

[147] Sundaramurthy V Barsacchi R Samusik N Marsico G Gilleron J Kalaidzidis I. Integration of Chemical and RNAi Multiparametric Profiles Identifies Triggers of Intracellular Mycobacterial Killing. Cell Host Microbe (2013) 13(2):129–42. 10.1016/j.chom.2013.01.008 23414754

[148] Stanley RE Ragusa MJ Hurley JH. The Beginning of the End: How Scaffolds Nucleate Autophagosome Biogenesis. Trends Cell Biol (2014) 24(1):73–81. 10.1016/j.tcb.2013.07.008 23999079 PMC3877172

[149] Dutta NK Pinn ML Karakousis PC. Metformin Adjunctive Therapy Does Not Improve the Sterilizing Activity of the First-Line Antitubercular Regimen in Mice. Antimicrobial Agents Chemother (2017) 61(8):e00652–17. 10.1128/AAC.00652-17 PMC5527622 28559262

[150] Bhat SA Iqbal IK Kumar A. Imaging the NADH: NAD+ Homeostasis for Understanding the Metabolic Response of Mycobacterium to Physiologically Relevant Stresses. Front Cell Infect Microbiol (2016) 6:145. 10.3389/fcimb.2016.00145 27878107 PMC5099167

[151] Piccaro G Pietraforte D Giannoni F Mustazzolu A Fattorini L. Rifampin Induces Hydroxyl Radical Formation in Mycobacterium Tuberculosis. Antimicrobial Agents Chemother (2014) 58(12):7527–33. 10.1128/AAC.03169-14 PMC4249506 25288092

[152] Alarcón De La Lastra C Villegas I. Resveratrol as an Antioxidant and Pro-Oxidant Agent: Mechanisms and Clinical Implications. Biochem Soc Trans (2007) 35(5):1156–60. 10.1042/BST0351156 17956300

[153] Kuršvietienė L Stanevičienė I Mongirdienė A Bernatonienė J. Multiplicity of Effects and Health Benefits of Resveratrol. Med (Kaunas) (2016) 52(3):148–55. 10.1016/j.medici.2016.03.003 27496184

[154] Liu M Wilk SA Wang A Zhou L Wang RH Ogawa W. Resveratrol Inhibits mTOR Signaling by Promoting the Interaction Between mTOR and DEPTOR. J Biol Chem (2010) 285(47):36387–94. 10.1074/jbc.M110.169284 PMC2978567 20851890

[155] Nakahira K Haspel JA Rathinam VAK Lee SJ Dolinay T Lam HC. Autophagy Proteins Regulate Innate Immune Responses by Inhibiting the Release of Mitochondrial DNA Mediated by the NALP3 Inflammasome. Nat Immunol (2011) 12(3):222–30. 10.1038/ni.1980 PMC3079381 21151103

[156] Zhou R Yazdi AS Menu P Tschopp J. A Role for Mitochondria in NLRP3 Inflammasome Activation. Nature (2011) 469(7329):221–6. 10.1038/nature09663 21124315

[157] Harris J Lang T Thomas JPW Sukkar MB Nabar NR Kehrl JH. Autophagy and Inflammasomes. Mol Immunol (2017) 86:10–5. 10.1016/j.molimm.2017.02.013 28249679

[158] Battah B Chemi G Butini S Campiani G Brogi S Delogu G. A Repurposing Approach for Uncovering the Anti-Tubercular Activity of FDA-Approved Drugs With Potential Multi-Targeting Profiles. Molecules (2019) 24(23):4373. 10.3390/molecules24234373 PMC6930672 31795400

[159] Castillo EF Dekonenko A Arko-Mensah J Mandell MA Dupont N Jiang S. Autophagy Protects Against Active Tuberculosis by Suppressing Bacterial Burden and Inflammation. Proc Natl Acad Sci USA (2012) 109(46):3168–76. 10.1073/pnas.1210500109 PMC3503152 23093667

[160] Griffith DE. Treatment of *Mycobacterium Avium* Complex (Mac). Semin Respir Crit Care Med (2018) 39(3):351–61. 10.1055/s-0038-1660472 30071550

[161] Luthra S Rominski A Sander P. The Role of Antibiotic-Target-Modifying and Antibiotic-Modifying Enzymes in *Mycobacterium Abscessus* Drug Resistance. Front Microbiol (2018) 9:2179. 10.3389/fmicb.2018.02179 30258428 PMC6143652

[162] Wu ML Aziz DB Dartois V Dick T. NTM Drug Discovery: Status, Gaps and the Way Forward. Drug Discovery Today (2018) 23(8):1502–19. 10.1016/j.drudis.2018.04.001 PMC6078814 29635026

[163] Chopra S Matsuyama K Hutson C Madrid P. Identification of Antimicrobial Activity Among FDA-approved Drugs for Combating *Mycobacterium Abscessus* and *Mycobacterium Chelonae* . J Antimicrob Chemother (2011) 66(7):1533–6. 10.1093/jac/dkr154 21486854

[164] Pang H Li G Zhao X Liu H Wan K Yu P. Drug Susceptibility Testing of 31 Antimicrobial Agents on Rapidly Growing Mycobacteria Isolates From China. BioMed Res Int (2015) 2015:419392. 10.1155/2015/419392 26351633 PMC4550772

[165] O’Riordan W Green S Overcash JS Puljiz I Metallidis S Gardovskis J. Omadacycline for Acute Bacterial Skin and Skin-Structure Infections. New Engl J Med (2019) 380(6):528–38. 10.1056/nejmoa1800170 30726689

[166] Celdrán A Esteban J Mañas J Granizo JJ. Wound Infections Due to Mycobacterium Fortuitum After Polypropylene Mesh Inguinal Hernia Repair. J Hosp Infect (2007) 66(4):374–7. 10.1016/j.jhin.2007.05.006 17655974

[167] Murillo J Torres J Bofill L Rios-Fabra A Irausquin E Istúriz R. Skin and Wound Infection by Rapidly Growing Mycobacteria: An Unexpected Complication of Liposuction and Liposculpture. The Venezuelan Collaborative Infectious and Tropical Diseases Study Group - Pubmed. Arch Dermatol (2000) 136(11):1347–52. 10.1001/archderm.136.11.1347 11074697

[168] Brickman M Parsa AA Parsa FD. Mycobacterium Cheloneae Infection After Breast Augmentation. Aesthet Plast Surg (2005) 29(2):116–8. 10.1007/s00266-004-0023-7 15759095

[169] Mauriello JA. Atypical Mycobacterial Infection of the Periocular Region After Periocular and Facial Surgery. Ophthalmic Plast Reconstructive Surg (2003) 19(3):182–8. 10.1097/01.IOP.0000064994.09803.CB 12918551

[170] Gravante G Caruso R Araco A Cervelli V. Infections After Plastic Procedures: Incidences, Etiologies, Risk Factors, and Antibiotic Prophylaxis. Aesthet Plast Surg (2008) 32(2):243–51. 10.1007/s00266-007-9068-8 18080159

[171] Goldman J Caron F De Quatrebarbes J Pestel-Caron M Courville P Doré MX. Infections From Tattooing: Outbreak of *Mycobacterium Chelonae* in France. BMJ (2010) 341:c5483. 10.1136/bmj.c5483 20978041

[172] Falkinham JO. Nontuberculous Mycobacteria From Household Plumbing of Patients With Nontuberculous Mycobacteria Disease. Emerg Infect Dis (2011) 17(3):419–24. 10.3201/eid1703.101510 PMC3166028 21392432

[173] Khan M Gibbons JL Ferrajoli A. Spotlight on Ibrutinib and its Potential in Frontline Treatment of Chronic Lymphocytic Leukemia. OncoTargets Ther (2017) 10:1909–14. 10.2147/OTT.S98689 PMC5384733 28408842

[174] Tran PN O’Brien S. The Safety of Bruton’s Tyrosine Kinase Inhibitors for the Treatment of Chronic Lymphocytic Leukemia. Expert Opin Drug Saf (2017) 16(9):1079–88. 10.1080/14740338.2017.1344213 28627951

[175] Dousa KM Babiker A Van Aartsen D Shah N Bonomo RA Johnson JL. Ibrutinib Therapy and *Mycobacterium Chelonae* Skin and Soft Tissue Infection. Open Forum Infect Dis (2018) 5(7):ofy168. 10.1093/ofid/ofy168 30090839 PMC6065501

[176] Fiorcari S Maffei R Audrito V Martinelli S Hacken E Zucchini P. Ibrutinib Modifies the Function of Monocyte/Macrophage Population in Chronic Lymphocytic Leukemia. Oncotarget (2016) 7(40):65968–81. 10.18632/oncotarget.11782 PMC5323207 27602755

[177] Li L Chen X Gu H. The Signaling Involving in Autophagy Machinery in Keratinocytes and Therapeutic Approaches for Skin Diseases. Oncotarget (2016) 7(31):50682–97. 10.18632/oncotarget.9330 PMC5226613 27191982

[178] Wang RC Levine B. Calcipotriol Induces Autophagy in Hela Cells and Keratinocytes. J Invest Dermatol (2011) 131(4):990–3. 10.1038/jid.2010.423 PMC3131686 21228817

[179] Ito K Koga M Shibayama Y Tatematsu S Nakayama J Imafuku S. Proactive Treatment With Calcipotriol Reduces Recurrence of Plaque Psoriasis. J Dermatol (2016) 43(4):402–5. 10.1111/1346-8138.13158 26434738

[180] Yuk JM Shin DM Lee HM Yang CS Jin HS Kim KK. Vitamin D3 Induces Autophagy in Human Monocytes/Macrophages Via Cathelicidin. Cell Host Microbe (2009) 6(3):231–43. 10.1016/j.chom.2009.08.004 19748465

[181] Fabri M Stenger S Shin DM Yuk JM Liu PT Realegeno S. Vitamin D is Required for IFN-γ-Mediated Antimicrobial Activity of Human Macrophages. Sci Trans Med (2011) 3(104):104ra102. 10.1126/scitranslmed.3003045 PMC3269210 21998409

[182] Vickers NJ. Animal Communication: When I’m Calling You, Will You Answer Too? Curr Biol (2017) 27(14):R713–5. 10.1016/j.cub.2017.05.064 28743020

[183] Martineau AR Honecker FU Wilkinson RJ Griffiths CJ. Vitamin D in the Treatment of Pulmonary Tuberculosis. J Steroid Biochem Mol Biol (2007) 103(3–5):793–8. 10.1016/j.jsbmb.2006.12.052 17223549

[184] Selvaraj P. Vitamin D, Vitamin D Receptor, and Cathelicidin in the Treatment of Tuberculosis. Vitam Horm (2011) 86:307–25. 10.1016/B978-0-12-386960-9.00013-7 21419277

[185] Liu PT Wheelwright M Teles R Komisopoulou E Edfeldt K Ferguson B. MicroRNA-21 Targets the Vitamin D-dependent Antimicrobial Pathway in Leprosy. Nat Med (2012) 18(2):267–73. 10.1038/nm.2584 PMC3274599 22286305

[186] Afsal K Selvaraj P Harishankar M. 1, 25-Dihydroxyvitamin D_3_ Downregulates Cytotoxic Effector Response in Pulmonary Tuberculosis. Int Immunopharmacol (2018) 62:251–60. 10.1016/j.intimp.2018.07.018 30032050

[187] Sanguinetti M Ardito F Fiscarelli E La Sorda M D’Argenio P Ricciotti G. Fatal Pulmonary Infection Due to Multidrug-Resistant Mycobacterium Abscessus in a Patient With Cystic Fibrosis. J Clin Microb (2001) 39(2):816–9. 10.1128/JCM.39.2.816-819.2001 PMC87830 11158161

[188] Nessar R Cambau E Reyrat JM Murray A Gicquel B. *Mycobacterium Abscessus*: A New Antibiotic Nightmare. J Antimicrob Chemother (2012) 67(4):810–8. 10.1093/jac/dkr578 22290346

[189] Williams A Sarkar S Cuddon P Ttofi EK Saiki S Siddiqi FH. Novel Targets for Huntington’s Disease in an mTOR-independent Autophagy Pathway. Nat Chem Biol (2008) 4(5):295–305. 10.1038/nchembio.79 18391949 PMC2635566

[190] Nowotarska SW Nowotarski K Grant IR Elliott CT Friedman M Situ C. Mechanisms of Antimicrobial Action of Cinnamon and Oregano Oils, Cinnamaldehyde, Carvacrol, 2,5-Dihydroxybenzaldehyde, and 2-Hydroxy-5-Methoxybenzaldehyde Against *Mycobacterium Avium* Subsp. *Paratuberculosis* (Map). Foods (2017) 6(9):72. 10.3390/foods6090072 PMC5615284 28837070

[191] Potoènjak I Gobin I Domitroviæ R. Carvacrol Induces Cytotoxicity in Human Cervical Cancer Cells But Causes Cisplatin Resistance: Involvement of MEK–ERK Activation. Phytother Res (2018) 32(6):1090–7. 10.1002/ptr.6048 29417642

[192] Spalletta S Flati V Toniato E Di Gregorio J Marino A Pierdomenico L. Carvacrol Reduces Adipogenic Differentiation by Modulating Autophagy and ChREBP Expression. PloS One (2018) 13(11):e0206894. 10.1371/journal.pone.0206894 30418986 PMC6231630

[193] Marini E Di Giulio M Ginestra G Magi G Di Lodovico S Marino A. Efficacy of Carvacrol Against Resistant Rapidly Growing Mycobacteria in the Planktonic and Biofilm Growth Mode. PloS One (2019) 14(7):e0219038. 10.1371/journal.pone.0219038 31260476 PMC6602199

[194] Brüning A Brem GJ Vogel M Mylonas I. Tetracyclines Cause Cell Stress-Dependent ATF4 Activation and mTOR Inhibition. Exp Cell Res (2014) 320(2):281–9. 10.1016/j.yexcr.2013.11.012 24280420

[195] Kaushik A Ammerman NC Martins O Parrish NM Nuermberger EL. *In Vitro* Activity of New Tetracycline Analogs Omadacycline and Eravacycline Against Drug-Resistant Clinical Isolates of *Mycobacterium Abscessus* . Antimicrob Agents Chemother (2019) 63(6):e470–19. 10.1128/aac.00470-19 PMC6535573 30962331

[196] Rodrigues L Wagner D Viveiros M Sampaio D Couto I Vavra M. Thioridazine and Chlorpromazine Inhibition of Ethidium Bromide Efflux in *Mycobacterium Avium* and *Mycobacterium Smegmatis* . J Antimicrob Chemother (2008) 61(5):1076–82. 10.1093/jac/dkn070 18310137

[197] Deshpande D Srivastava S Musuka S Gumbo T. Thioridazine as Chemotherapy for *Mycobacterium Avium* Complex Diseases. Antimicrob Agents Chemother (2016) 60(8):4652–8. 10.1128/aac.02985-15 PMC4958214 27216055

[198] Seervi M Rani A Sharma AK Santhosh, Kumar TR. ROS Mediated ER Stress Induces Bax-Bak Dependent and Independent Apoptosis in Response to Thioridazine. Biomed Pharmacother (2018) 106:200–9. 10.1016/j.biopha.2018.06.123 29960166

[199] Chu CW Ko HJ Chou CH Cheng TS Cheng HW Liang YH. Thioridazine Enhances P62-Mediated Autophagy and Apoptosis Through Wnt/β-Catenin Signaling Pathway in Glioma Cells. Int J Mole Sci (2019) 20(3):473. 10.3390/ijms20030473 PMC6386927 30678307

[200] Bermudez LE Kolonoski P Wu M Aralar PA Inderlied CB Young LS. Mefloquine Is Active In Vitro and In Vivo Against Mycobacterium Avium Complex. Antimicrob Agents Chemother (1999) 43(8):1870–4. 10.1128/aac.43.8.1870 PMC89383 10428905

[201] Shin JH Park SJ Jo YK Kim ES Kang H Park JH. Suppression of Autophagy Exacerbates Mefloquine-mediated Cell Death. Neurosci Lett (2012) 515(2):162–7. 10.1016/j.neulet.2012.03.040 22465322

